# NIR‐Triggered Upconversion‐Perovskite Heterostructures for Non‐Genetic, Implant‐Free Optoelectronic Neuromodulation

**DOI:** 10.1002/advs.202513844

**Published:** 2025-11-23

**Authors:** Luyue Jiang, Chenguang Ma, Yiping Zhao, Jiazhi Li, Gen Li, Shuang Jin, Haoyang Su, Ye Tian, Yingkang Yang, Yunfu Luo, Lei Huang, Peijie Chen, Yiming Gao, Yi Wei, Yike Xiang, Lunming Qin, Kaihuan Zhang, Yifei Ye, Pengyi Tang, Liuyang Sun

**Affiliations:** ^1^ State Key Laboratory of Transducer Technology Shanghai Institute of Microsystem and Information Technology Chinese Academy of Sciences Shanghai 200050 China; ^2^ 2020 X‐Lab Shanghai Institute of Microsystem and Information Technology Chinese Academy of Sciences Shanghai 200050 China; ^3^ School of Integrated Circuits University of Chinese Academy of Sciences Beijing 100049 China; ^4^ College of Electronics and Information Engineering Shanghai University of Electric Power Shanghai 201306 China; ^5^ National Key Laboratory of Materials for Integrated Circuits Shanghai Institute of Microsystem and Information Technology Chinese Academy of Sciences Shanghai 200050 China

**Keywords:** deep brain regions;non‐genetic, non‐invasion, optoelectronic neuromodulation, upconversion‐perovskite heterostructure

## Abstract

Optoelectronic neuromodulation has transformed neuroscience research and holds great promise for treating neurological disorders. However, conventional optoelectronic methods rely on ultraviolet/visible light, which poorly penetrates tissue and typically necessitates surgically implanted optical fibers for deep‐brain stimulation. Here, a heterostructure is presented that integrates near‐infrared (NIR)‐excitable upconversion nanoparticles (UCNPs) and broadband‐absorbing CsPbBr_3_ perovskite quantum dots (QDs). This nanostructure converts deeply penetrating 980 nm NIR light into localized electrical stimuli, enabling immediate and precise modulation of neuronal activity without implants. In vitro, NIR illumination of this heterostructure reliably increases the firing rate of wild‐type dopaminergic (DA) neurons in acute brain slices. Importantly, in vivo, transcranial NIR stimulation of the heterostructure in the secondary motor cortex (M2) and ventral tegmental area (VTA) modulates neuronal activity, triggers turning behavior, and promotes dopamine release. Moreover, it exhibits negligible neuroinflammation and structural stability in brain tissue over at least four weeks. By integrating a stable heterostructure for efficient NIR‐driven photocurrent generation, the method offers a non‐genetic, minimally invasive platform for precise neuromodulation in wild‐type animals.

## Graphical abstract

The Single‐Nanostructured Optoelectronic Vehicle for neuromodulation Activation (SNOVA) establishes a paradigm for non‐genetic, implant‐free neuromodulation. By integrating NIR‐excitable UCNPs with broadband‐absorbing perovskite QDs, SNOVA efficiently converts deeply penetrating light into localized electric fields that modulate neuronal ion dynamics, trigger behavioral responses, and enable precise, minimally invasive deep‐brain modulation.

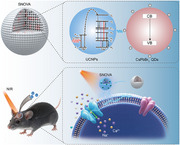

## 1 Introduction

Precise neuromodulation in living animals is essential for investigating neural function and developing therapies for neurological disorders.^[^ ^1^, ^2^, ^3^, ^4^, ^5^ ^]^ Electrical stimulation has long been the predominant technique, with its efficacy varying depending on whether electrodes are positioned intracellularly or extracellularly. Intracellular approaches, which involve direct access to the neuronal interior, offer high efficacy by modulating membrane potential dynamics with minimal current due to close proximity to ion channels. For instance, the patch‐clamp method^[^ ^6^ ^]^ enables precise single‐cell modulation in vivo but is limited to individual neurons. More recently, microhole and microwire electrode arrays^[^ ^7^, ^8^ ^]^ have shown potential for intracellular access to large numbers of neurons, though their application remains confined to in vitro settings.

In contrast, extracellular methods, which position electrodes outside the neuronal membrane, offer significant advantages for in vivo applications, enabling neuromodulation in freely behaving animals across broader brain regions. Deep brain stimulation (DBS),^[^ ^9^ ^]^ widely used for treating neurological disorders, exemplifies this strength by providing effective, broad‐scale modulation with large electrodes. Advances in microelectronics have further enhanced this capability, with high‐density probes like Neuropixels and Neuralink^[^ ^10^, ^11^ ^]^ enabling stable, long‐term recordings from hundreds of neurons individually, and ultraflexible endovascular probes^[^ ^12^ ^]^ facilitating brain recording through micrometer‐scale vasculature. However, these extracellular approaches demand higher energy inputs to overcome tissue impedance and vascular barriers, posing challenges for effective stimulation.

As an alternative, optogenetics provides high‐efficiency stimulation by expressing light‐sensitive opsins within neurons, facilitating membrane potential changes with relative ease. This method also enables access to large numbers of neurons in vivo, enhancing its utility in research. However, optogenetics requires genetic modification, limiting its applicability across species and in clinical settings. Additionally, its reliance on blue light, which has limited tissue penetration, often necessitates implanted optical fibers, restricting stimulation range and introducing risks of tissue damage.

To address these limitations, NIR light‐based neuromodulation has gained increasing attention. NIR light operates within the biological tissue optical window,^[^ ^13^, ^14^ ^]^ enabling transcranial energy delivery to subcortical regions without implants.^[^ ^15^, ^16^, ^17^, ^18^ ^]^ Current NIR light‐based neuromodulation strategies primarily rely on nanotransducers that convert NIR into secondary energy forms—such as visible light, heat, or mechanical forces—to modulate neuronal activity in either transgenic or wild‐type mice.^[^ ^19^, ^20^, ^21^ ^]^ These approaches are generally classified as optogenetic,^[^ ^15^, ^22^, ^23^ ^]^ optothermal,^[^ ^24^, ^25^, ^26^, ^27^, ^28^ ^]^ or optomechanical stimulation.^[^ ^29^, ^30^, ^31^, ^32^ ^]^ For instance, in optogenetics, UCNPs are employed to convert NIR into visible light to activate genetically encoded, light‐sensitive ion channels such as channelrhodopsins.^[^ ^15^, ^33^, ^34^ ^]^ Another example is the use of radioluminescent nanoparticles that emit ≈610 nm light in situ upon X‐ray excitation, improving tissue penetration for high‐precision, wireless optogenetics.^[^ ^35^ ^]^ Although these NIR‐based approaches offer effective modulation, they often require genetic modification or involve thermal and mechanical stimuli that pose risks of tissue damage, limiting their translational and clinical applicability.

More recently, our group demonstrated the feasibility of an NIR‐optoelectronic strategy by converting NIR into extracellular electrical stimulation in deep brain regions.^[^ ^36^ ^]^ This strategy offers greater versatility than optogenetic or thermal methods because electrical signals inherently couple to neuronal activity.^[^ ^37^, ^38^, ^39^ ^]^ The majority of photovoltaic materials, which are employed to convert light into electrical stimuli in the neuromodulation regime, respond to ultraviolet or visible light^[^ ^40^, ^41^, ^42^, ^43^ ^]^ lacking sufficient tissue penetration.^[^ ^44^ ^]^ To expand the spectrum to the NIR range, we physically mixed UCNPs and photovoltaic nanoparticles, where NIR was first upconverted to blue emission and subsequently converted into localized photocurrents, demonstrating a proof of concept for non‐genetic and implant‐free DBS.^[^ ^36^ ^]^

Despite the effectiveness of the hybrid system, the overall efficiency in converting NIR into electrical signals was vulnerable to the stochastic nature of the mixing process of the two components, resulting from the uneven dispersion and unstable interface. Indeed, their distinct morphologies and densities led to different sedimentation rates, resulting in phase separation and inconsistent component ratios. These limitations underscore the need for a single nanostructure capable of both upconversion and optoelectronic conversion under NIR illumination.

**Figure 1:**
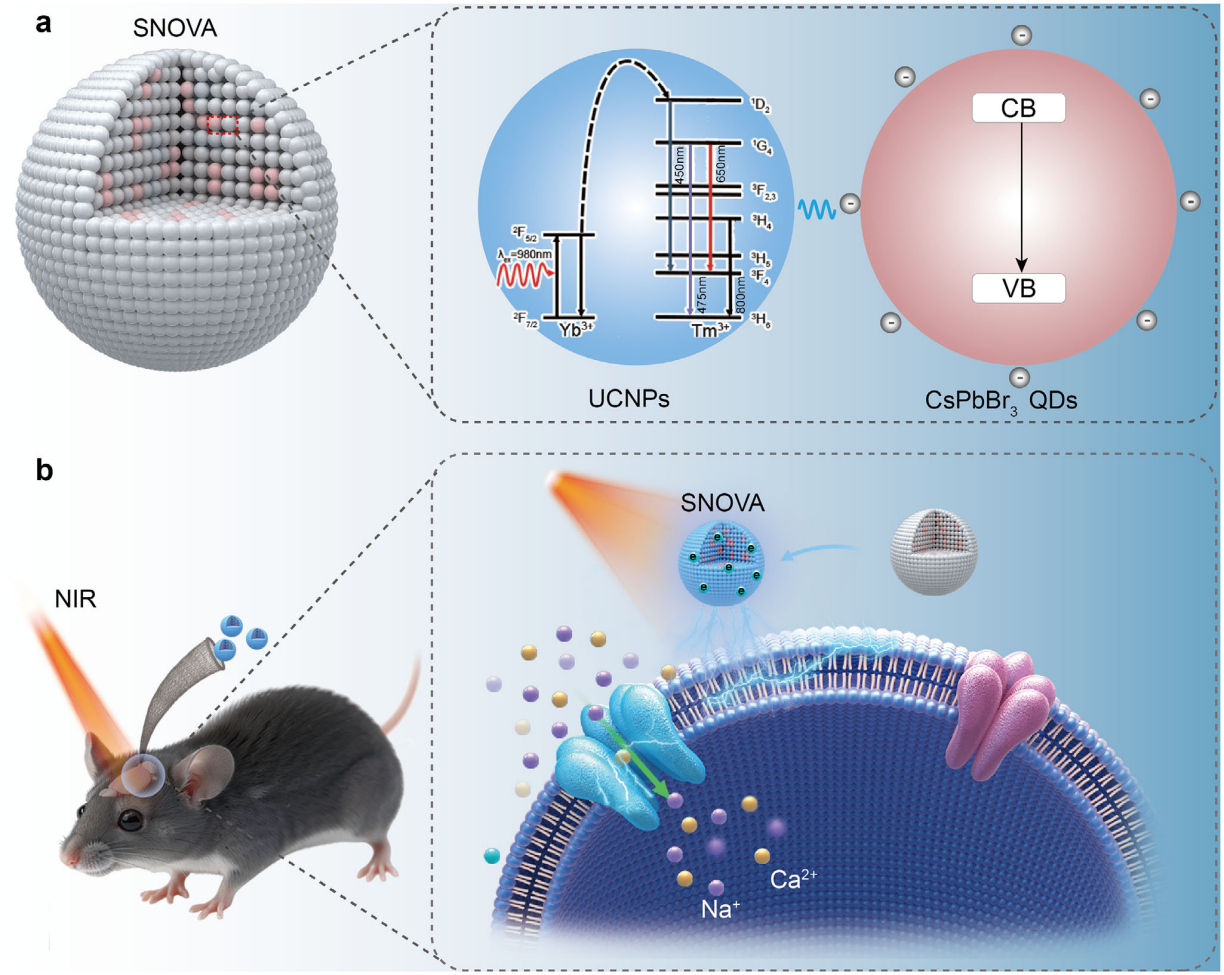
Non‐invasive and nongenetic neuromodulation via NIR‐triggered cascaded photoelectrics in SNOVA. a) Schematic of the SNOVA heterostructure and photoelectric activation mechanism. The UCNPs matrix absorbs 980 nm NIR light and emits visible photons, which are subsequently absorbed by embedded CsPbBr_3_ nanocrystals and converted into localized photoelectric responses. b) Illustration of spatially localized neuromodulation in cortical and deep brain regions. Transcranial NIR illumination activates SNOVA at the injection site, generating localized photoelectric signals that modulate neuronal activity without the need for genetic modification or implanted devices.

In this work, we developed a heterostructure by co‐precipitating UCNPs and perovskite QDs as a novel Single‐Nanostructured Optoelectronic Vehicle for neuromodulation Activation (SNOVA) as illustrated in **Figure** **1**. These components respectively enable NIR‐to‐visible upconversion^[^ ^34^, ^45^, ^46^ ^]^ and efficient light‐to‐electric conversion.^[^ ^47^, ^48^, ^49^ ^]^ Leveraging its structural integrity and optoelectronic synergy, SNOVA enables stable, minimally invasive, and non‐genetic neuromodulation under NIR illumination. Here, we demonstrate that SNOVA enables robust neuromodulation both in vitro and in vivo. In acute brain slices, NIR illumination of SNOVA induced immediate action potentials in wild‐type DA neurons, confirming its ability to directly evoke neuronal firing in vitro. For in vivo validation, we successfully stimulated the M2 and the VTA, serving as representative examples of cortical and subcortical brain regions. In freely behaving mice, transcranial NIR stimulation of the SNOVA‐injected M2 region elicited pronounced turning behavior as early as two days post‐injection, supporting cortical circuit activation. In the VTA, transcranial NIR stimulation of the SNOVA‐injected VTA region activated DA neurons, as confirmed by real‐time electrochemical dopamine detection as early as seven days post‐injection. Behavioral assays further revealed place preference in a Y‐maze following NIR stimulation, providing functional evidence of deep‐brain regions modulation. Immunofluorescence staining showed elevated c‐Fos expression near the injection sites, indicating neuronal activation at the cellular level. Importantly, SNOVA exhibited stable optical and electrical performance and minimal immunogenicity over four weeks. These results establish SNOVA as a non‐genetic, minimally invasive platform for DBS, offering a distinct mechanism of action through NIR‐triggered photocurrents rather than optical or thermal gating.

## 2 Results and Discussion

### 2.1 Synthesis and Characterization of SNOVA

**Figure 2:**
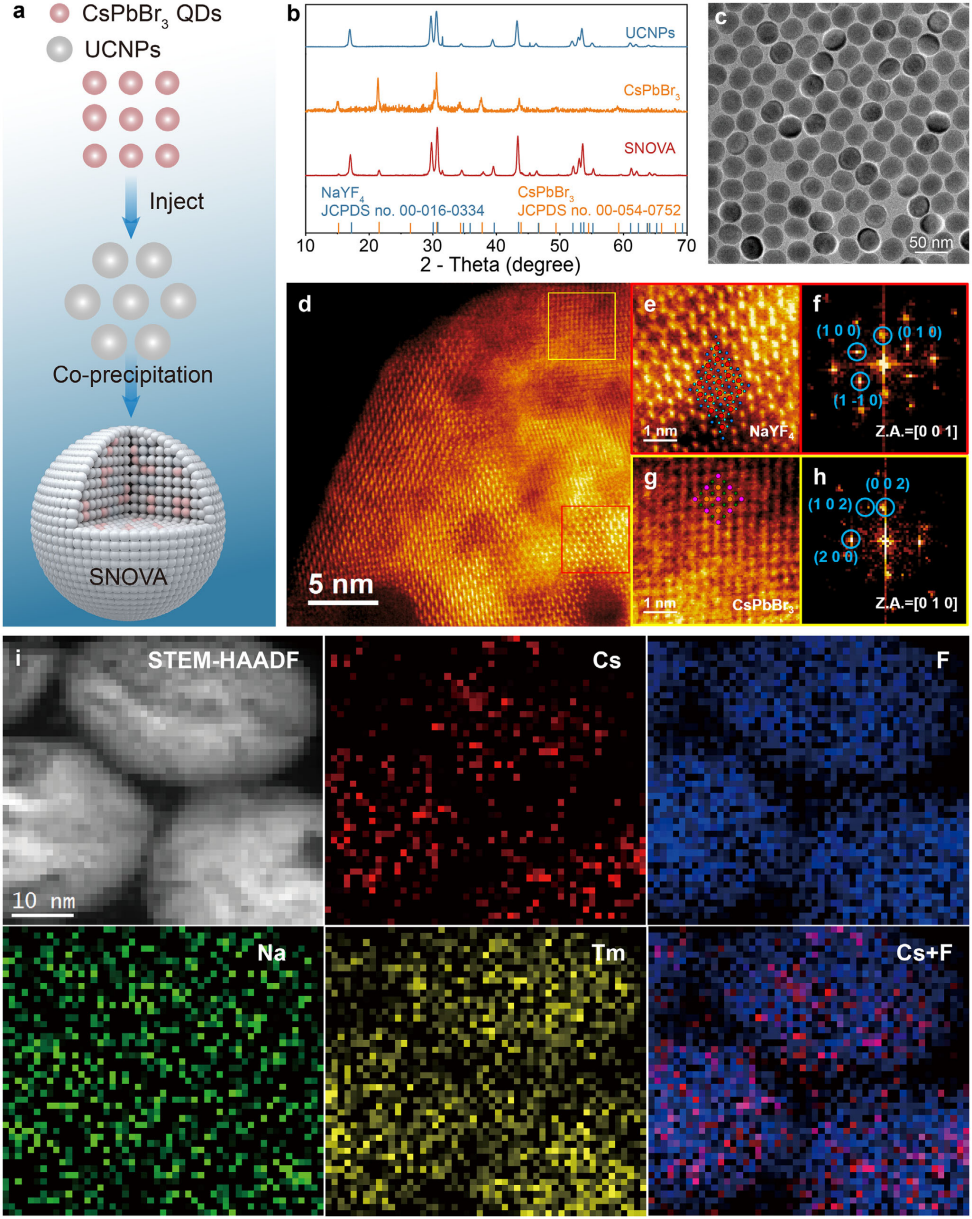
Structural and compositional analysis of SNOVA. a) Schematic of the SNOVA design, showing CsPbBr_3_ nanocrystals embedded in a NaYF_4_:Yb/Tm UCNPs matrix for cascaded NIR‐to‐photocurrent conversion. b) XRD patterns of UCNPs, CsPbBr_3,_ and SNOVA. c) TEM images of SNOVA. d) STEM‐HAADF images of SNOVA after Wiener filtering. e) Enlarged image of the red square area marked in (d) with overlapping atomic models of hexagonal NaYF_4_:Yb/Tm UCNPs. f) Corresponding FFT patterns of the orange square area marked in (d). g) Enlarged image of the yellow square area marked in (d) with overlapping atomic models of cubic CsPbBr_3_. h) Corresponding FFT patterns of the yellow square area marked in (d). i) STEM‐HAADF image and EELS elemental maps of SNOVA, including Cs (Red, CsPbBr_3_), F (Blue, UCNPs), Na (Green, UCNPs), and Tm (Yellow, UCNPs).

To enable minimally invasive, non‐genetic neuromodulation via NIR light, we designed and synthesized SNOVA heterostructures that couple upconversion and photoinduced electronic processes in a single nanoscale platform. Specifically, SNOVA heterostructures were synthesized via a co‐precipitation method by co‐precipitating CsPbBr_3_ nanocrystals with lanthanide‐doped UCNP precursors, leading to partially embedded CsPbBr_3_ domains within the NaYF_4_:Yb/Tm matrix.^[^ ^50^, ^51^ ^]^ Specifically, cubic‐phase CsPbBr_3_ nanocrystals were first synthesized using a standard hot‐injection protocol^[^ ^52^ ^]^ and were then subjected to a precursor solution containing Yb^3^⁺, Y^3^⁺ and Tm^3^⁺ to initiate UCNPs growth. During high‐temperature reaction, the smaller CsPbBr_3_ nanocrystals were incorporated into the UCNPs matrix, forming a composite nanostructure (**Figure** **2**a). Following the synthesis of the SNOVA heterostructure, the nanoparticles were treated with a mixed solution of ethanol (5 mL) and HCl (5 mL, 2 M) under sonication for 5 min. The resulting oleic acid (OA)‐free, water‐soluble SNOVA nanoparticles were collected by centrifugation at 7000 rpm for 5 min. The surface hydrophilicity was evaluated by comparing static water contact angles of SNOVA with and without OA removal, as presented in Figure S1 (Supporting Information). The contact angles for OA‐removed SNOVA (14.81° and 15.92°) were markedly lower than those for OA‐coated SNOVA (64.22° and 67.76°), demonstrating excellent hydrophilicity and compatibility with aqueous solvents.

Transmission electron microscopy (TEM) revealed uniform spherical morphology of SNOVA with an average particle diameter of ≈45 nm (Figure 2c; Figures S2 and S3, Supporting Information). X‐ray diffraction (XRD) analysis showed multiphase composition, with cubic CsPbBr_3_ with *Pm*3_*m* space group (JCPDS no. 00‐054‐0752) coexisting with hexagonal NaYF_4_ (JCPDS no. 00‐016‐0334) matrix (Figure 2b).

To directly observe the structure of the SNOVA, aberration‐corrected scanning transmission electron microscopy‐high angle annular dark field (STEM‐HAADF) imaging was performed (Figure 2d–h; Figure S4, Supporting Information). The atomically resolved HAADF‐STEM images of SNOVA reveal sharp structural contrast between NaYF_4_:Yb/Tm and CsPbBr_3_ domains. For NaYF_4_:Yb/Tm, enlarged views projected along the [001] zone axis exhibit precise atomic registry with the corresponding hexagonal structural models (Figure 2e). The fast Fourier transform (FFT) pattern of NaYF_4_:Yb/Tm exhibited ordered characteristic reflections of the FFT spots of (100), (010), and (1–10) lattice planes along the [001] zone axis, confirming its hexagonal structure (Figure 2f). For CsPbBr_3_, the lattice along the [010] zone axis shows spacing and symmetry consistent with cubic CsPbBr_3_, also in excellent agreement with the simulated model (Figure 2g). The FFT pattern of CsPbBr_3_ further shows reflections corresponding to (200), (102), and (002) lattice planes along the [010] zone axis, confirming its cubic structure (Figure 2h).

Furthermore, elemental mapping was carried out using STEM‐electron energy loss spectroscopy (EELS) (Figure 2i; Figure S5, Supporting Information). The elemental distributions of F, Tm, and Na correspond to NaYF_4_:Yb/Tm UCNPs, whereas Cs indicates the presence of CsPbBr_3_. The spatial distribution confirms that NaYF_4_:Yb/Tm and CsPbBr_3_ domains are closely integrated and unevenly embedded within the heterojunction. According to the elemental distributions of Cs and F, the heterostructure is mainly composed of NaYF_4_:Yb/Tm UCNPs, while CsPbBr_3_ accounts for a smaller proportion. Together, the atomically resolved HAADF‐STEM micrographs and EELS elemental maps provide direct and unambiguous evidence for the coexistence of hexagonal NaYF_4_:Yb/Tm UCNPs and cubic CsPbBr_3_ domains, thereby substantiating the successful fabrication of the SNOVA heterostructures. Additionally, X‐ray photoelectron spectroscopy (XPS) results further confirmed this integration (Figure S6, Supporting Information).

To determine optimal composition, SNOVA heterostructures were synthesized by varying the input molar ratios of UCNPs:CsPbBr_3_ nanocrystals during the preparation process, and their structural integrity was evaluated by TEM. Structural collapse was observed when the added CsPbBr_3_ nanocrystal content exceeded an input molar ratio of 1:0.7, indicating over‐saturation of perovskite domains within the matrix (Figures S2 and S3, Supporting Information). An input molar ratio of 1:0.5 yielded an intact, monodisperse heterostructure with robust morphology and was selected for all subsequent experiments.

### 2.2 Optoelectronic Performance of SNOVA

To assess the capacity of SNOVA to transduce NIR light into neuromodulatory electrical signals, we characterized its optoelectronic response using absorption spectrum, photoluminescence (PL) spectrum, steady‐state surface photovoltage (SPV) spectrum, and photocurrent measurements.

**Figure 3:**
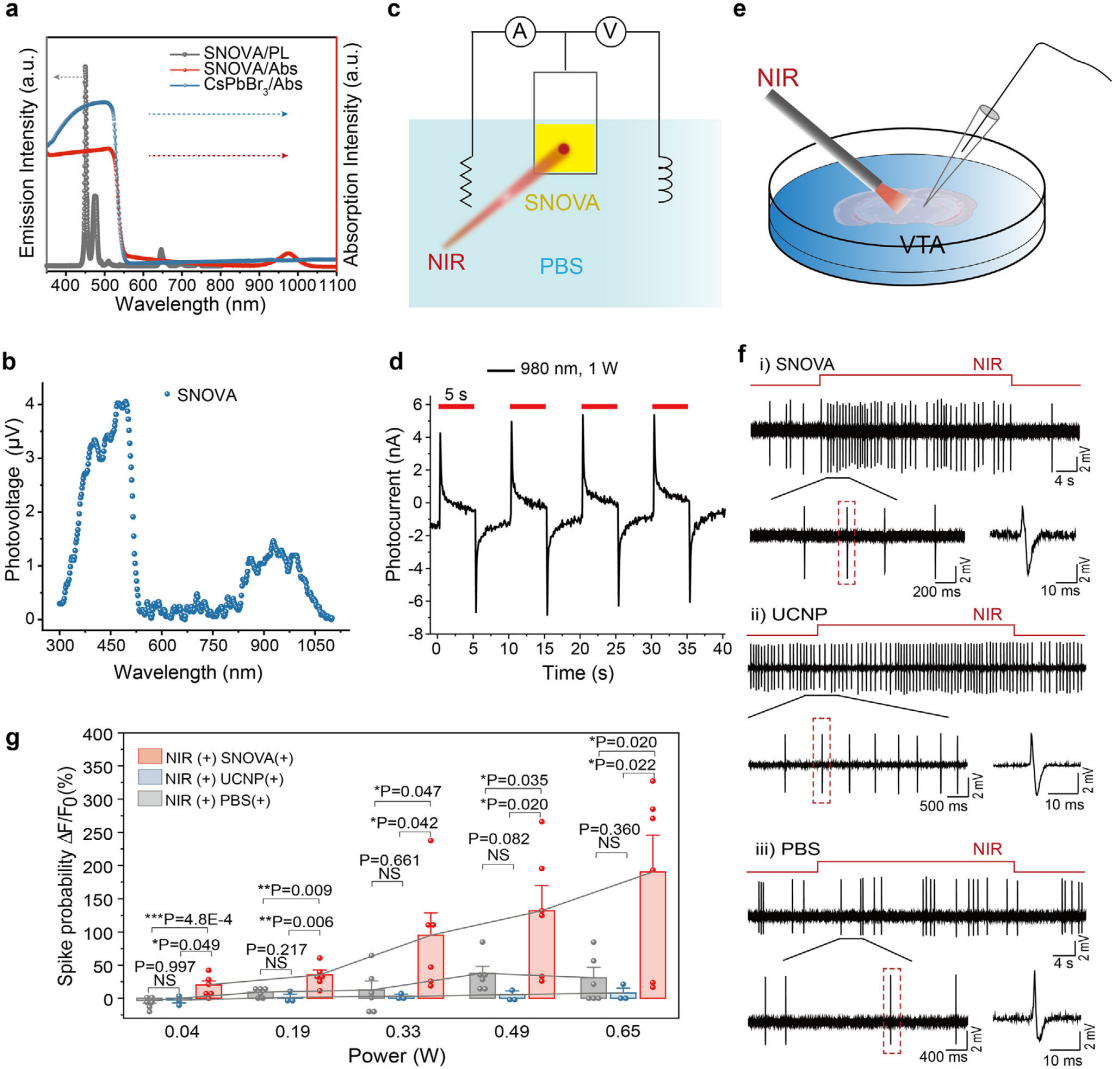
Photoelectric and neurophysiological characterization of SNOVA. a) Absorption spectra of SNOVA and CsPbBr_3_ nanocrystals, and PL spectrum of SNOVA excited by 980 nm laser. b) SPV spectrum of SNOVA. c) Schematic diagram of the three‐electrode system for photocurrent measurements. d) Time‐dependent photocurrent (I‐t) of SNOVA under 980 nm illumination (1 W) measured in PBS (pH 7.4). e) Schematic of the patch‐clamp setup for neuronal recordings. f) (i) Cell‐attached recordings of firing activity in VTA dopamine neurons before and after NIR stimulation in an acute brain slice treated with SNOVA. (ii) Cell‐attached recordings of firing activity in VTA dopamine neurons before and after NIR stimulation in an acute brain slice treated with UCNP. (iii) Cell‐attached recordings of firing activity in VTA dopamine neurons before and after NIR stimulation in an acute brain slice treated with PBS. NIR stimulation was performed using a 980 nm pulsed laser (0.5 Hz, 300 ms pulse width, 0.33 W). g) Laser power‐dependent modulation of firing rate in VTA dopamine neurons under NIR stimulation (n = 6 cells for SNOVA group, n = 3 cells for UCNP group, n = 6 cells for PBS group). Data are shown as mean ± SEM, with each point representing an independent trial; All groups followed a normal distribution, and a two‐sample *t*‐test was performed to calculate P values.

First, the absorption spectrum of the SNOVA reveals a dual profile: a narrow absorption peak at 980 nm attributable to the UCNPs and a broadband absorption band spanning 300–550 nm associated with the CsPbBr_3_ nanocrystals (**Figure** **3**a). This dual absorption confirms the effective integration of the two components and highlights the heterostructure's potential for remote neuromodulation in deep brain regions, as 980 nm light falls within the tissue‐transparency window of the electromagnetic spectrum.^[^ ^53^ ^]^

To evaluate the intermediate emission characteristics in the cascade photoelectric process, the PL spectrum of SNOVA was recorded under 980 nm laser excitation. Strong emissions were observed at 450 and 475 nm, attributed to UCNP‐mediated upconversion (Figure 3a; Figure S7, Supporting Information). This upconversion process involves the absorption of 980 nm photons by UCNPs (Figure 1a), where the unique 4f energy levels of lanthanide ions (e.g., Yb^3+^ and Tm^3+^, with transitions such as ^2^F_7/2_ → ^2^F_5/2_ and ^1^G_4_ → ^3^H_6_) facilitate the emission of visible light.^[^ ^54^ ^]^ These emission bands spectrally align with the absorption range of CsPbBr_3_ nanocrystals, affording the interband transition in CsPbBr_3._^[^ ^55^ ^]^

To verify that light interaction within SNOVA effectively induces photoresponsive potential changes, we measured the SPV response across a spectral range of 300 to 1100 nm (Figure 3b). The SPV spectrum of the SNOVA revealed two pronounced peaks: one at 980 nm, corresponding to UCNPs absorption, and another spanning 300–550 nm, matching the absorption band of CsPbBr_3_. For comparative insight, SPV spectra of pure CsPbBr_3_, NaYF_4_ UCNPs, and bare fiuorine‐doped tin oxide (FTO) were also acquired (Figure S8, Supporting Information). While NaYF_4_ UCNPs and FTO showed negligible responses across this range, CsPbBr_3_ displayed a strong signal below 500 nm, with no significant response at 980 nm.^[^ ^56^ ^]^ The unique 980 nm peak in SNOVA is thus attributed to the upconversion process, wherein NaYF_4_ UCNPs absorb 980 nm NIR light and emit visible photons (450/475 nm), which are subsequently absorbed by embedded CsPbBr_3_ nanocrystals, driving the observed surface photovoltage under NIR illumination.

To establish that SNOVA produces functionally relevant photoelectric outputs in a physiological context, we measured photocurrent responses in a three‐electrode system using phosphate‐buffered saline (PBS) as the electrolyte (Figure 3c). SNOVA nanostructures were drop‐cast onto FTO working electrodes, and photocurrent was recorded under 980 nm laser excitation. Photocurrent measurements revealed that the SNOVA produced a peak current of 6.15 nA under 1 W laser power (Figure 3d), whereas pure FTO and FTO coated with NaYF_4_ UCNPs showed negligible responses under the same conditions (Figure S9, Supporting Information). This output reflects efficient photon harvesting and charge generation across the heterostructure, confirming SNOVA's ability to convert NIR light into stable electrical signals under bio‐relevant conditions.

We note that the insulating nature of NaYF_4_ UCNPs may potentially reduce charge transfer from CsPbBr_3_ to the external brain tissue. However, EELS mapping reveals that CsPbBr_3_ nanocrystals are randomly distributed, with some located near the surface and separated by only a few nanometers of NaYF_4_, as shown in Figure S5 (Supporting Information). This thin insulating layer supports possible direct electron tunneling. Furthermore, the internal electric fields generated within the SNOVA heterostructure can drive ionic concentration changes in adjacent tissue, enabling neuronal excitation without requiring direct charge injection into the neurons.

### 2.3 In Vitro Neuromodulation Mediated by SNOVA

To evaluate the photoelectric neuromodulation capacity of SNOVA, we performed ex vivo brain slice electrophysiological experiments (Figure 3e). A 3 µL PBS suspension of SNOVA (100 mg mL^−1^) was delivered to the VTA of mouse brain slices, allowing the heterostructures to diffuse and settle within the tissue. Following stabilization, we employed the cell‐attached patch‐clamp technique to record the electrical activity of DA neurons during 980 nm NIR stimulation.

In SNOVA‐treated slices, 980 nm NIR stimulation (300 ms pulses at 0.5 Hz, 0.33 W) induced a reliable increase in neuronal firing. Neuronal activity was recorded using the cell‐attached patch‐clamp technique over a 50‐s window, with stimulation initiated at the 10th second and maintained for 30 s (Figure 3f). Across all tested neurons, firing rate increased during NIR illumination. In a representative recording (Figure 3f(i)), the firing rate increased from 0.52 Hz (baseline) to 1.66 Hz during NIR stimulation. This effect was observed in repeated recordings from the same neuron and across six recorded neurons (Figure S10, Supporting Information). In contrast, control slices applied pure UCNPs (Figure 3f(ii)) and PBS only (Figure 3f(iii)) showed no observable change in firing rate under identical conditions (Figure S11, Supporting Information). These findings support the ability of SNOVA to modulate neuronal excitability via photoelectrochemical conversion in wild‐type tissue.

To assess the dose‐response relationship, we quantified firing modulation as the normalized firing rate, calculated as (F–F_0_)/F_0_, where F and F_0_ represent the firing frequencies during and before illumination, respectively. Power‐dependent recordings revealed a monotonic increase in the normalized firing rate among DA neurons adjacent to SNOVA, with mean values of 20.14 ± 6.23 (P < 0.01, two‐sample t‐test), 35.54 ± 7.38 (P < 0.01, two‐sample t‐test), 95.06 ± 32.79 (P < 0.05, two‐sample t‐test), 132.13 ± 37.67 (P < 0.05, two‐sample t‐test), and 190.19 ± 55.54 (P < 0.05, two‐sample t‐test) at 0.04 W, 0.19 W, 0.33 W, 0.49 W, and 0.65 W, respectively (Figure 3g). Control slices showed minimal variation across power levels, with the mean values of all groups remaining below 38. Together, these results demonstrate a clear dose–response relationship and highlight the tuneable photoelectric characteristics of SNOVA as a driver of neuronal modulation.

### 2.4 In Vivo Neuromodulation Mediated by SNOVA in the Mouse Motor Cortex

Having established that SNOVA enables neuromodulation via NIR‐induced photoelectric conversion in vitro, we next explored its neuromodulatory potential in vivo by focusing on the motor cortex—a region central to voluntary movement coordination and widely used in cortical stimulation studies.^[^ ^57^, ^58^, ^59^, ^60^ ^]^ We hypothesized that 980 nm transcranial illumination could activate SNOVA particles in the motor cortex by inducing localized optoelectronic effects, thereby modulating neuronal activity and triggering turning behavior in freely moving mice.^[^ ^61^, ^62^ ^]^

**Figure 4:**
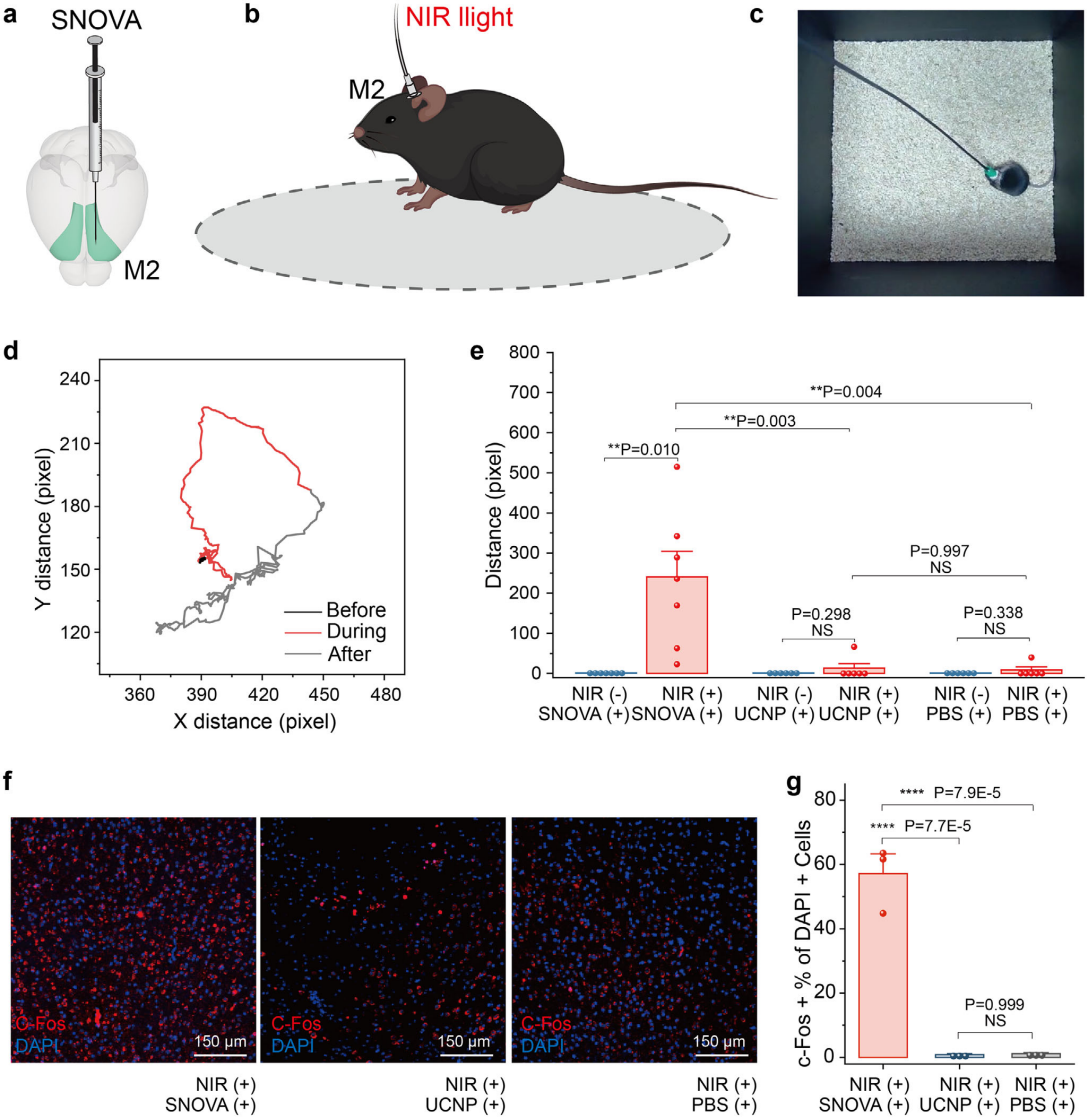
SNOVA‐mediated neuromodulation in the M2 region. a) Schematic diagram of SNOVA injection into the motor cortex. b) Schematic illustration of SNOVA activating motor neurons for turning behavior via a flexible, non‐invasive fiber. c) An illustrative image depicting the experimental setup used for motor behavior regulation via SNOVA. d) A representative locomotion trajectory of an SNOVA‐treated mouse before (black line), during (red line), and after (grey line) NIR stimulation of the M2 motor cortex. e) Statistical analysis of linear displacements of mice at under different experimental conditions. n = 7 mice for the NIR (+) SNOVA (+) group and n = 6 mice for NIR (+) UCNP (+) group and NIR (+) PBS (+) group. Data are shown as mean ± SEM, with each point representing an independent trial; P values were calculated by one‐way ANOVA with Tukey's post‐hoc comparison test. f) Confocal images of the M2 region following transcranial NIR stimulation under different conditions. g) Percentage of c‐Fos‐positive cells in the DAPI‐stained cell population under the three conditions shown in (g) (n = 3 mice each). P values were calculated by one‐way ANOVA with Tukey's post‐hoc comparison test.

To test this, we injected 3 µL of SNOVA suspension (100 mg mL^−1^ in PBS) into the left M2 region of mice (AP: +1 mm, ML: +0.78 mm, DV: −1 mm; n = 6) (**Figure** **4**a). Control groups received PBS or UCNPs alone (n = 6 each). All animals exhibited full postoperative recovery within 24 h. Mice were subjected to transcranial NIR stimulation (4 Hz, 150 ms pulse width, 0.8 W peak power, 10 s duration) during behavioral quiescence (Figure 4b). A head‐mounted flexible optical fiber (0.9 mm outer diameter) enabled unrestricted movement while maintaining light delivery (Figure 4c). The locomotion trajectory of an SNOVA‐treated mouse is shown in Figure 4d, illustrating movement before (black), during (red), and after (grey) NIR stimulation of the M2 motor cortex. NIR stimulation was manually triggered upon visual confirmation that the mice were at rest to minimize spontaneous locomotion and ensure a stable baseline. During the stimulation period, the mouse exhibited markedly increased movement. Specifically, the average displacement of SNOVA‐treated mice during stimulation (240.8 ± 64.0 pixels) was substantially greater than that before stimulation (1.2 ± 0.6 pixels). For comparison, locomotion trajectories of PBS‐treated and UCNP‐treated control mice are provided in Figure S12 (Supporting Information), both showing minimal movement changes under identical stimulation conditions.

To quantify group‐level behavioral responses during stimulation, we analyzed linear displacements recorded in the 10‐s NIR illumination period across experimental and control groups. SNOVA‐treated mice exhibited significantly greater displacement during NIR stimulation compared to PBS‐treated (8.9 ± 18.4 pixels) and UCNP‐treated (13.6 ± 26.3 pixels) mice (P < 0.01, one‐way ANOVA with Tukey post hoc; Figure 4e). In contrast, mice in the control groups showed no significant difference in displacement during NIR stimulation (P = 0.997, two‐sample t‐test). These data indicate that SNOVA‐mediated photoelectrochemical activation of the motor cortex is associated with enhanced locomotor activity relative to control conditions.

To further validate SNOVA‐mediated neuronal activation in the M2 motor cortex, we performed quantitative immunohistochemical analysis of c‐Fos expression, a well‐established immediate‐early gene marker that peaks 1–2 h post‐neuronal activation.^[^ ^63^, ^64^ ^]^ Specifically, three experimental cohorts were analyzed: NIR(+)/SNOVA (+) mice (n = 3), along with control groups of NIR(+)/UCNP(+) (n = 3) and NIR(+)/PBS(+) mice (n = 3). Confocal microscopy quantification revealed a ≈45‐fold increase in c‐Fos+ nuclei density in SNOVA‐treated mice compared to both control groups (p < 0.0001, one‐way ANOVA with Tukey post‐hoc), with activated neurons constituting 57.12 ± 3.56% of DAPI+ cells in the target region (Figure 4f,g). Notably, control groups exhibited baseline c‐Fos expression levels indistinguishable from non‐stimulated conditions, confirming the essential role of both NIR illumination and the SNOVA nanocomplex in achieving specific neuronal activation. This histochemical evidence further confirms that SNOVA‐mediated phototransduction effectively modulates cortical circuitry through optoelectronic stimulation.

### 2.5 In Vivo Neuromodulation Mediated by SNOVA in VTA

We further explored whether the SNOVA‐based photoelectric stimulation system could achieve neuromodulation in deep brain regions. The VTA was selected due to its deep location within the brain and its critical role in the reward circuitry, as well as its association with behaviors linked to major depressive disorder.^[^ ^65^ ^]^ While current stimulation strategies such as optogenetics,^[^ ^15^, ^66^ ^]^ electrical,^[^ ^67^ ^]^ and thermal^[^ ^61^ ^]^ modalities can activate this region, non‐genetic and non‐invasive optoelectric stimulation of the VTA in wild‐type mice has not yet been explored.

**Figure 5:**
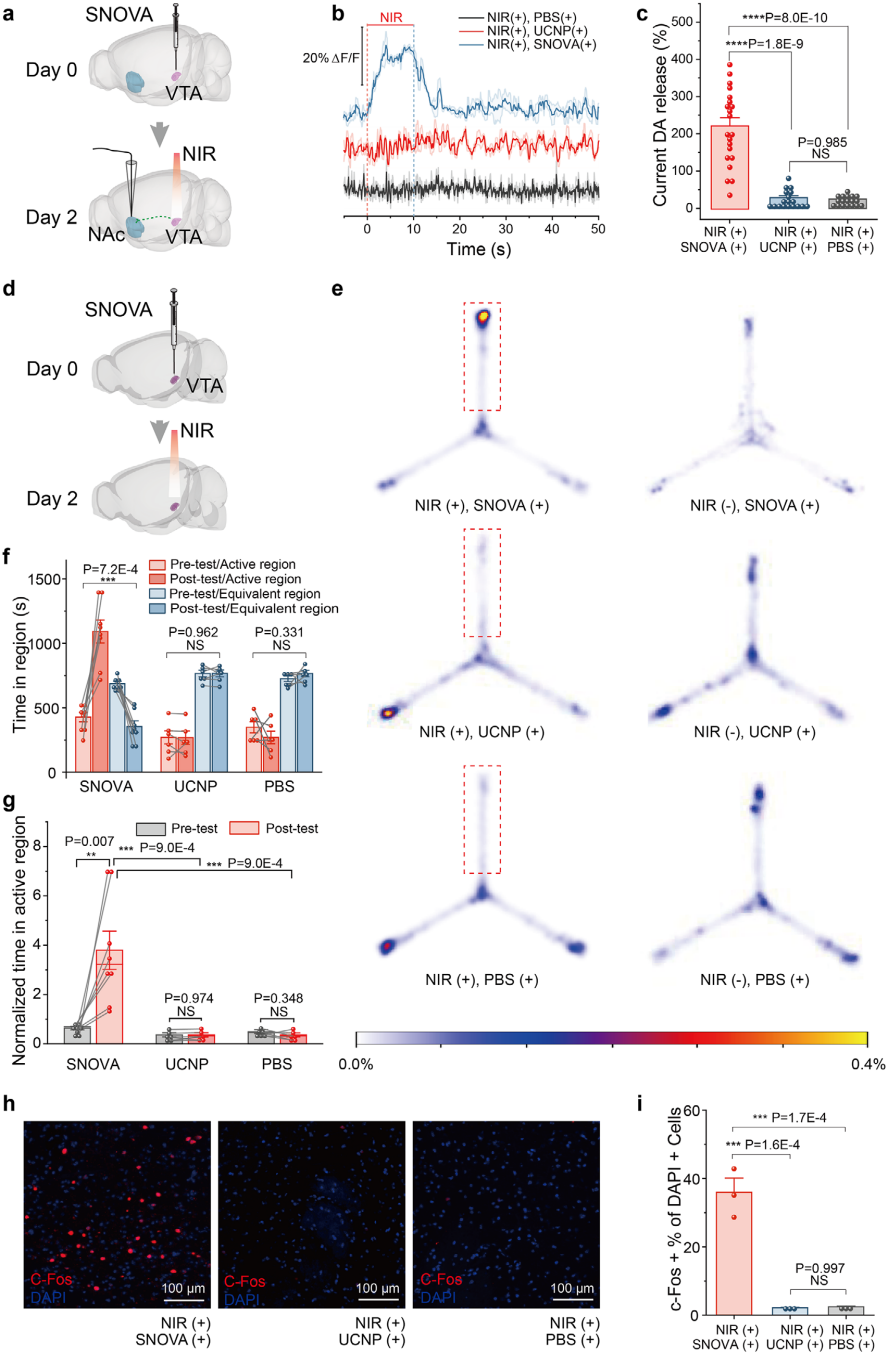
SNOVA neuromodulation of the VTA region in vivo. a) Schematic of the electrochemical setup for recording DA transients in the NAc during transcranial NIR stimulation of the VTA in vivo. b) Averaged curves of relative DA concentration change in the NAc in response to transcranial VTA stimulation under different experimental conditions. Each curve represents the mean of three repeated trials from one mouse in the corresponding group (NIR(+)/SNOVA(+), NIR(+)/UCNP(+), and NIR(+)/PBS(+)), with shaded areas representing SEM (standard error of the mean). Vertical dashed lines mark the onset (red) and offset (blue) of the 10‐s NIR stimulation window. c) Statistics analysis of cumulative DA release within 30 s after initiating transcranial stimulation under the three groups illustrated in (b). Each data point represents a single stimulation trial (3 trials per mouse). n = 7 mice for NIR(+)/SNOVA(+); n = 6 mice for NIR(+)/UCNP(+) and NIR(+)/PBS(+) groups. Data are presented as mean ± SEM. P values were calculated using one‐way ANOVA with Tukey's post hoc test. d) Schematic diagram of SNOVA injection into VTA. e) Representative post‐test heat maps showing the time of travel of mice under different experimental conditions. Red dashed squares indicate NIR irradiated regions. f) Statistical analysis of time spent in the NIR illuminated region, and average time spent in the other regions under different experimental conditions. n = 8 mice for the NIR (+)/SNOVA(+) group and n = 6 mice for all other groups. Data are shown as mean ± SEM, with each point representing an independent trial; All groups followed a normal distribution, and a paired *t*‐test was performed to calculate P values. g) Statistical analysis of the normalized time spent in the NIR illuminated region under different experimental conditions. n = 8 mice for the NIR (+)/SNOVA(+) group and n = 6 mice for all other groups. Data are shown as mean ± SEM, with each point representing an independent trial; All groups followed a normal distribution, and a paired *t*‐test was performed to calculate P values. P values of three post‐test groups were calculated by one‐way ANOVA with Tukey's post‐hoc comparison test. h) Confocal images of the VTA area following transcranial NIR stimulation under different conditions. i) Percentage of c‐Fos‐positive cells in the DAPI‐stained cell population under the three conditions shown in (d) (n = 3 mice each). The data are expressed as mean ± SEM, with each point representing an individual trial from a total of N = 3 trials; P values were calculated by one‐way ANOVA with Tukey's post‐hoc comparison test.

We first evaluated the real‐time excitatory effects of SNOVA on DA neurons in the VTA using electrochemical analysis. SNOVA was stereotaxically injected into the VTA, and dopamine dynamics in the nucleus accumbens (NAc)—a downstream projection area—were continuously monitored via a three‐electrode setup (**Figure** **5**a, see Experimental section). The transient fluctuations in NAc dopamine levels reflect VTA neuron activity, underscoring their therapeutic relevance for major depressive disorder.^[^ ^68^ ^]^ By measuring changes in the amplitude of the dopamine oxidation peak current via an electrochemical workstation, we quantified the dopamine release induced by optoelectrical stimulation. In mice pretreated with SNOVA, a 10‐s NIR stimulation (140 ms pulses, 1 Hz, 1.5 W peak power) induced DA release lasting 15 s, peaking ≈10 s post‐illumination (Figure 5b). Each curve in Figure 5b represents the mean of three trials from a single mouse, showing consistent DA elevation in the SNOVA group. In contrast, control groups injected with UCNPs or PBS exhibited no detectable DA changes during the same stimulation window. Comparative analysis revealed substantially enhanced cumulative dopamine release in SNOVA‐treated mice versus control cohorts, demonstrating the system's effectiveness in photoelectric neuromodulation (p < 0.0001, one‐way ANOVA with Tukey post‐hoc; Figure 5c).

We next investigated the neuromodulatory effects of SNOVA on the VTA using a conditioned place preference test. SNOVA were injected into the VTA of mice, followed by contextual training in a Y‐maze (Figure 5d). During training, transcranial NIR stimulation (980 nm, 150 ms pulses at 4 Hz, 0.8 W peak power) was applied to the VTA. After three consecutive days of photoelectric stimulation training, SNOVA‐injected mice exhibited a strong preference for the NIR‐illuminated arm of the Y‐maze, spending nearly twice as much time there during the post‐test compared to the pre‐test (p < 0.001, one‐way ANOVA with Tukey post hoc; Figure 5e,f). In contrast, no significant difference in arm preference was observed in mice injected with UCNPs or PBS before and after NIR training (Figure 5f,g). These findings confirm that SNOVA enables specific and effective optoelectronic modulation of VTA neurons in freely moving mice, without the need for implanted optical fibers or electrodes.

To confirm SNOVA‐induced activation in the deep brain region, we quantified c‐Fos expression in the VTA, a region central to DA signaling. Specifically, three experimental groups were analyzed: NIR(+)/SNOVA(+) mice (n = 3), along with control groups of NIR(+)/UCNP(+) (n = 3) and NIR(+)/PBS(+) mice (n = 3). Confocal microscopy quantification revealed a more than 14‐fold increase in c‐Fos+ nuclei density in SNOVA‐treated mice compared to both control groups (p < 0.001, one‐way ANOVA with Tukey post‐hoc), with activated neurons constituting 35.95 ± 4.23% of DAPI+ cells in the target region (Figure 5h,i). Notably, control groups exhibited baseline c‐Fos expression levels indistinguishable from non‐stimulated conditions, confirming the essential role of both NIR illumination and the SNOVA nanocomplex in achieving specific neuronal activation. This histochemical evidence further confirms that SNOVA‐mediated phototransduction effectively modulates deep brain regions through optoelectronic stimulation.

### 2.6 Stability and Biocompatibility Evaluation of SNOVA

To comprehensively evaluate the structural, optoelectronic, and biological robustness of SNOVA, we systematically assessed its stability and biocompatibility under in vitro and in vivo conditions.

To assess SNOVA's structural and optoelectronic robustness, SNOVA samples were incubated in deionized water under ambient conditions and analyzed at days 0, 7, 14, and 35 using TEM and photocurrent measurements. We observed no clear changes in morphology or photocurrent response (Figures S2c, S13 and S14, Supporting Information), confirming SNOVA's long‐term structural and optoelectronic stability in aqueous media.

SNOVA samples were also immersed in PBS (37 °C) to further evaluate their stability under physiological conditions. ICP‐MS analysis showed that the relative proportions of all metal elements (Yb, Na, Y, Pb, Cs, Tm, and Br) maintained similar levels over 14 days (Figure S15, Supporting Information). STEM‐EDX mapping confirmed the persistent presence of all elements after 21 days (Figure S16, Supporting Information). However, a gradual decrease in particle size was observed during prolonged immersion, with an overall reduction of ≈12.8% after 21 days (Figure S17, Supporting Information), suggesting partial surface degradation. These results indicate that SNOVA maintains overall compositional integrity and structural robustness under physiological conditions, while undergoing mild surface degradation over time.

To assess the effectiveness and safety of SNOVA‐mediated neuromodulation, we employed SNOVA's intrinsic fluorescence tracking and immunohistochemistry for Iba1, GFAP, and Caspase‐3 to detect microglial activation, astrocytic response, and apoptosis, respectively.

**Figure 6:**
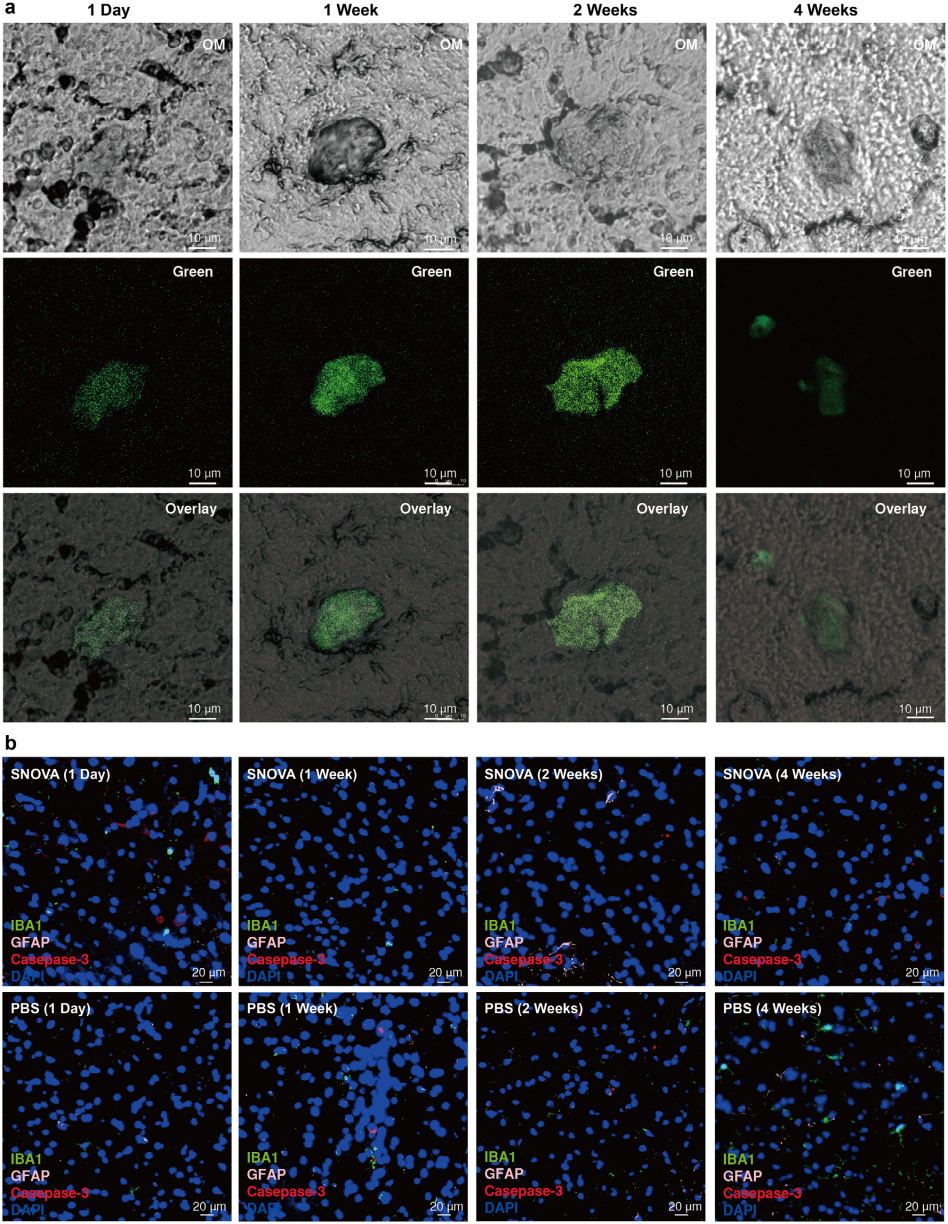
In vivo stability and biocompatibility of SNOVA nanostructures. a) Optical microscopy (OM), microscopic confocal fluorescence (green), and overlay images of brain slices collected at 0 day, 1 week, 2 weeks, and 4 weeks following stereotaxic injection of SNOVA into the VTA. Microscopic confocal images were acquired under 405 nm excitation and 510 nm emission. b) Representative confocal images showing immunofluorescence staining of the VTA at 1 day, 1 week, 2 weeks, and 4 weeks post‐injection for SNOVA‐injected (top row) and PBS‐injected control mice (bottom row). Sections were labeled with Iba1 (green, microglia), GFAP (pink, astrocytes), Caspase‐3 (red, apoptosis), and DAPI (blue, nuclei).

We assessed the in vivo effectiveness of SNOVA by injecting it into the VTA of mice and tracking its intrinsic fluorescence under 405 nm excitation with 510 nm emission. Brain tissues were collected at 1 day, 1 week, 2 weeks, and 4 weeks post‐injection, and 60‐µm‐thick frozen sections were imaged using confocal fluorescence microscopy (**Figure** **6**a). Fluorescence signals remained spatially confined within ≈40 µm radius of the injection site with minimal diffusion, and signal intensity remained stable over four weeks, with only a modest decline observed at day 28. These results indicate the high spatial resolution and consistent presence of SNOVA in vivo.

Biocompatibility was assessed by immunofluorescence analysis of Iba1, GFAP, and Caspase‐3 expression in mice injected with SNOVA or PBS into the VTA. Brain sections were collected at 1 day, 1 week, 2 weeks, and 4 weeks post‐injection. Immunofluorescence staining was performed for Iba1, GFAP, Caspase‐3, and DAPI to assess local immune and apoptotic responses.^[^ ^69^, ^70^ ^]^ From a full view of a representative brain section, a clear injection tract outlined by microglia was observed, confirming accurate delivery into the VTA (Figure S18a, Supporting Information). In the enlarged views, there were no apparent increases in Iba1, GFAP, or Caspase‐3 expression in SNOVA‐injected mice compared to PBS controls at any time point (Figure 6b; Figure S18b, Supporting Information). Moreover, the density of glial and apoptotic cells remained stable over the 4‐week period (Figure 6b). These findings indicate that SNOVA elicits minimal inflammatory or cytotoxic responses over four weeks.

To further assess the impact of SNOVA injection on animal well‐being, we monitored body weight and locomotor activity in SNOVA‐treated and PBS‐control mice over a one‐month period (see Experimental Section for details). Both groups displayed a consistent increase in body weight, with no significant differences observed (Figure S19a, Supporting Information). Likewise, total distance travelled in the OFT remained comparable between groups across all time points (Figure S19b, Supporting Information), suggesting that SNOVA administration at the tested dose had no detectable adverse effects on general health or locomotion.

Taken together, these results demonstrate that SNOVA maintains optoelectronic stability, preserves localized and persistent presence in the brain, and exhibits minimal inflammatory or cytotoxic responses, without detectable effects on body weight or behavior over four weeks. However, we note that SNOVA showed slightly gradual dissolution over time, indicating that Pb^2+^ ion release may occur over extended periods, highlighting the importance of further long‐term biosafety evaluation.

## 3 Conclusion

In summary, SNOVA is established as a minimally invasive and transgene‐free neuromodulation platform that directly converts NIR light into bioactive electrical signals. By integrating UCNPs with perovskite QDs into a single heterostructure, SNOVA enables cascade optoelectronic conversion—first transforming 980 nm light into visible emissions via UCNPs, then generating photocurrent through the photoactive perovskite domain. This mechanism bypasses the need for genetic modification or thermal mediation and offers a distinct pathway to neuromodulate wild‐type neurons with high spatial and temporal precision.

We demonstrated that SNOVA could reliably stimulate both superficial and deep brain regions in vivo using only transcranial NIR light. In the cortex, SNOVA‐mediated stimulation triggered behaviorally relevant turning movements in freely moving mice, accompanied by robust upregulation of c‐Fos expression in the motor cortex. In the VTA, SNOVA activation evoked dopamine release, as detected electrochemically in the nucleus accumbens, and induced place preference in a Y‐maze paradigm—demonstrating neuromodulatory engagement of midbrain reward circuitry. Notably, all experiments were conducted without implanted optical fibers or viral vectors, highlighting the system's non‐invasive and gene‐independent nature.

Beyond functional activation, SNOVA exhibited robust in vivo stability and safety. Fluorescence tracking showed that the nanostructures remained spatially confined with minimal diffusion for at least 4 weeks, and retained optical and photoelectric functionality. Immunohistochemical staining for microglial (Iba1), astrocytic (GFAP), and apoptotic (Caspase‐3) markers revealed no significant immune response or cytotoxicity compared to PBS controls, supporting favorable biocompatibility for long‐term brain integration.

Compared with existing technologies such as optogenetics or magnetothermal stimulation,^[^ ^71^, ^72^ ^]^ SNOVA offers a simplified and flexible alternative. Specifically, optogenetics relies on virus‐mediated gene delivery and extended expression timelines,^[^ ^1^, ^15^, ^67^ ^]^ limiting its applicability across species. In contrast, SNOVA enables immediate neuromodulation in wild‐type animals, enhancing its translational potential. Optogenetic stimulation typically requires a light power density of 10–100 mW mm^−2^, substantially lower than the ≈1 W mm^−2^ used for SNOVA, reflecting the intracellular advantage of opsins, which directly modulate membrane potential with high efficiency. SNOVA, however, generates localized electric fields to drive ionic currents extracellularly, necessitating higher power due to tissue impedance. This efficiency gap motivates future efforts to enhance SNOVA's performance through band‐engineering of upconversion nanoparticles and photovoltaic materials, aiming to reduce power requirements and improve clinical viability.

Nevertheless, several aspects warrant further optimization. First, the optical penetration remains limited. Although 980 nm excitation lies within the biological optical window, it is subject to scattering and absorption by intact skull and deep brain tissue, allowing effective penetration primarily in thin‐skull mouse models. Second, the long‐term stability could be further improved. While SNOVA exhibits moderate stability over 21 days in PBS with slight dissolution leading to a ≈12.8% reduction in particle diameter (Figure S15, Supporting Information), this gradual degradation may result in trace Pb^2+^ release under prolonged physiological exposure. Given the well‐documented neurotoxicity of lead, even at low concentrations, this represents a critical limitation for clinical translation. Future efforts should prioritize the development of lead‐free perovskite alternatives or robust surface passivation strategies to eliminate Pb leakage while preserving optoelectronic performance. Third, the cellular selectivity remains to be enhanced. Compared with genetic optogenetic approaches, SNOVA achieves spatial selectivity mainly through the localized nanoparticle distribution (≈40 µm radius) and confined effective electric field (≈50 µm) near the injection site, which helps reduce off‐target activation but do not yet confer cell‐type specificity.

Future work will focus on extending excitation wavelengths toward the second NIR window for deeper tissue penetration. The development of lead‐free perovskite analogs or robust encapsulation strategies could further enhance biosafety.^[^ ^73^ ^]^ Incorporating targeting ligands or engineering selective delivery vehicles may enable cell‐type‐specific control while maintaining the genetic independence of the platform. Additionally, although stereotaxic injection provides localized delivery, less invasive approaches (such as systemic administration with blood‐brain barrier modulation or targeted nanocarriers) would increase translational feasibility.^[^ ^74^ ^]^

Looking ahead, integrating semiconductor heterostructure into the SNOVA design could enable bidirectional control—both excitation and inhibition—via tailored illumination. Combined with emerging delivery strategies, SNOVA has the potential to evolve into a precision neuromodulation tool suitable for both experimental and therapeutic applications.^[^ ^39^ ^]^

In summary, SNOVA represents a new class of optical neuromodulators that combine the advantages of deep tissue penetration, non‐invasiveness, and direct electrical stimulation without genetic modification. Its ability to engage functionally distinct brain circuits and maintain long‐term bioactivity in vivo opens new opportunities in neuroscience research and non‐invasive neurotherapeutics.

## 4 Experimental Section

### Chemicals and Materials

Cesium carbonate (Cs_2_CO_3_, 99.9%), lead bromide (PbBr_2_, 99.9%), yttrium(III) chloride hexahydrate (YCl_3_·6H_2_O, 99.9%), ytterbium(III) chloride hexahydrate (YbCl_3_·6H_2_O, 99.9%), thulium(III) chloride hexahydrate (TmCl_3_·6H_2_O, 99.9%), ammonium fluoride (NH_4_F, 98%), sodium hydroxide (NaOH, 97%), oleic acid (OA, 90%), 1‐octadecene (ODE, 90%), oleylamine (OM,90%), cyclohexane (99.5%), methanol (99.5%) and ethanol (99.8%) were purchased from Aladdin and without further purification.

### Synthesis of CsPbBr_3_ Nanocrystals

Cs_2_CO_3_ (0.08, 0.11, 0.19, 0.26 mmol), ODE (3 mL), and OA (3 mL) were loaded into a 50 mL three neck flask and dried under argon atmosphere at 120 °C for 1 h, until all the Cs_2_CO_3_ reacted with OA. PbBr_2_ (0.2, 0.3, 0.5, 0.7 mmol), ODE (10 mL), OM (5 mL), and OA (5 mL) were loaded into a 50 mL flask and heated to 120 °C under argon atmosphere. After complete dissolution of the PbBr_2_, the temperature was raised to 150 °C and the preheated Cs‐oleate solution was quickly injected in for reaction of 10 s, cooled by an ice water bath. The CsPbBr_3_ were purified by cyclohexane and separated by centrifugation at 7000 rpm for 3 min.

### Synthesis of UCNPs

First, the uniform core nanoparticles NaYF_4_:30%Yb/0.5%Tm were synthesized by the coprecipitation method. A typical procedure is as follows: YCl_3_·6H_2_O (0.695 mmol), YbCl_3_·6H_2_O (0.3 mmol) and TmCl_3_·6H_2_O (0.005 mmol) were added into a 50 mL three‐necked flask containing 6 mL OA and 15 mL 1‐octadecene (ODE). The mixture was first heated to 150 °C under argon for 30 min to form a transparent solution. The solution was cooled down to room temperature, and 8 mL of a methanol solution containing NaOH (2.5 mmol) and NH_4_F (4 mmol) was slowly dropped into the flask and stirred for 30 min. Then, the solution was heated to 80 °C and maintained for 20 min to evaporate methanol. The solution was heated to 150 °C to evaporate water. Subsequently, the solution was heated to 300 °C and maintained for 1.5 h under argon atmosphere. After cooling down to room temperature, the resulting products were precipitated by ethanol and collected by centrifugation at 7000 rpm for 3 min. The precipitate was then purified with methanol and ethanol three times, and finally dispersed in cyclohexane for further use.

Second, the NaYF_4_ shell was synthesized. 1 mmol YCl_3_·6H_2_O were added to a 50 mL flask containing 6 mL of OA and 15 mL of ODE. The mixture was heated to 150 °C under argon for 30 min to form a transparent solution and then cooled down to room temperature, and 8 mL of a methanol solution containing NaOH (2.5 mmol) and NH_4_F (4 mmol) was slowly dropped into the flask and stirred for 20 min. Then, the solution was heated to 80 °C to evaporate methanol. The solution was heated to 150 °C for 10 min. Last, the reaction solution was cooled down to room temperature as NaYF_4_ shell precursors.

Third, NaYF_4_:30%Yb/0.5%Tm @NaYF_4_ core‐shell nanoparticles were synthesized. The epitaxial growth of NaYF_4_:Yb/Tm@NaYF_4_ to form a core‐shell structure was realized by a hot‐injection method. The nanoparticles NaYF_4_:Yb/Tm (0.4 mmol) core samples were added to a 50 mL flask containing 5 mL of OA and 10 ml of ODE. The mixture was heated to 300 °C. After that, 2 mL of NaYF_4_ shell precursors were injected into the reaction mixture step by step with an injection rate of 0.2 mL every 3 min. Upon completion of the injection, the reaction solution was cooled to room temperature, and the resulting NaYF_4_:Yb/Tm@NaYF_4_ core–shell nanoparticles were washed with cyclohexane, ethanol, and methanol.

### Synthesis of Heterostructured SNOVA

The SNOVA nanoparticles were synthesized according to a previously reported method.^48^ A typical procedure is as follows: YCl_3_·6H_2_O (0.695 mmol), YbCl_3_·6H_2_O (0.3 mmol) and TmCl_3_·6H_2_O (0.005 mmol) were added into a 100 mL of three‐necked flask containing 12 mL of oleic acid (OA) and 30 mL of 1‐octadecene (ODE). The mixture was first heated to 150 °C under argon atmosphere for 30 min to form a transparent solution. The solution was cooled down to room temperature. And 8 mL of a methanol solution containing NaOH (2.5 mmol) and NH_4_F (4 mmol) was slowly dropped into the flask and stirred for 30 min. Then, the solution was heated to 130 °C to evaporate methanol and water. The purified of CsPbBr_3_ (0.2, 0.3, 0.5, 0.7 mmol) were injected into the solution. Subsequently, the solution was heated to 300 °C and maintained for 1 h under argon atmosphere. After cooling down to room temperature, the resulting products were precipitated by ethanol and collected by centrifugation at 7000 rpm for 3 min. The precipitate was then purified with methanol and ethanol three times, and finally dispersed in cyclohexane for further use. To obtain ligand‐free, water‐soluble SNOVA nanoparticles, the as‐synthesized OA‐coated SNOVA nanoparticles were treated with a mixed solution of ethanol (5 mL) and HCl (5 mL, 2 M) under sonication for 5 min, following a standard protocol.^[^ ^75^ ^]^ The ligand free water‐soluble SNOVA nanoparticles were collected by centrifugation at 7000 rpm for 5 min.

### Structural and Optical Characterization

Drop the SNOVA solution dispersed in cyclohexane onto a carbon film‐coated copper grid (Beijing Zhongjingkeyi Technology Co., Ltd., China) and dry at room temperature for 48 h. Afterwards, TEM, STEM and elemental mapping of SNOVA were measured at 200 kV on a JEM‐2100F electron microscope (JEOL, Japan) and a HF5000 electron microscopy (Hitachi High‐Tech, Japan). The statistics of the nanocrystal sizes were calculated based on the size of ≈100 nanoparticles using Nano Measurer software. The X‐ray diffractograms of SNOVA nanoparticles powders, UCNPs powder and CsPbBr_3_ powder were performed on a powder diffractometer D8 ADVANCE (Bruker, Germany) with monochromatized Cu Kα radiation. Meanwhile, the UV–vis‐NIR absorption spectra of nanoparticles were measured by a Cary 5000 spectrophotometer (Agilent, America) and the measured range was 300–1100 nm. The PL spectra of SNOVA nanoparticles and UCNPs were obtained using an Ideaoptics Instruments confocal fluorescence spectrometer (300–800 nm) at room temperature. The excitation wavelength and the power of the laser were 980 nm and 0.98 W, respectively. The acquisition time was 100 milliseconds, and the imaging magnification of the objective lens used to acquire the PL spectra is 10X. The PL spectra of CsPbBr_3_ nanocrystals were obtained using a FLS 1000 photoluminescence spectrometer (Edinburgh Instruments, England) with excitation wavelength of 380 nm. STEM‐HAADF imaging and STEM‐EELS mapping were conducted using a double‐aberration‐corrected transmission electron microscope operating at 300 kV, achieving a spatial resolution of 82 pm. The microscope was equipped with an integrated GIF Continuum ER 1065 EELS system. The HAADF detector collection semi‐angles were set in the range of 45–180 mrad. To minimize background noise, the STEM‐HAADF images were processed using a Wiener filter within the STEM cell framework.^[^ ^28^, ^76^ ^]^ Image analysis was performed using Gatan Digital Micrograph software. For evaluation of the structural and photoelectric stability of SNOVA in aqueous media, SNOVA samples were immersed in DI water at room temperature for up to 35 days. Aliquots were collected after 7, 14, and 35 days for TEM imaging and photocurrent measurements. Morphological characterization was conducted using TEM (HF 5000, Hitachi), and photocurrent responses were recorded using a three‐electrode configuration under 980 nm illumination (1 W, PBS electrolyte, pH 7.4). For characterization of SNOVA stability under physiological conditions, SNOVA samples were immersed in PBS at 37 °C to simulate in vivo physiological conditions. Elemental distributions were examined by STEM‐EDX (HF 5000 and SPETRA 300) to assess compositional uniformity and stability after 0, 7, 14, and 21 days of immersion. Elemental contents (Yb, Na, Y, Pb, Cs, Tm, Br) were quantified using ICP‐MS (Agilent 7500CE) after 0 and 14 days of immersion.

### Photoelectrochemical Performance

Five milligrams of SNOVA heterostructures were dispersed in 1 mL of cyclohexane. Using a micropipette, 50 µL of the mixed solution (corresponding to 0.25 mg of SNOVA heterostructure) were deposited onto one side of the conductive glass (FTO). The samples were air‐dried in a fume hood to prepare the SNOVA heterostructure films. Subsequently, the films were placed in a laboratory oven at 200 °C for 120 min to relieve any stress. Transient photocurrent measurements were conducted using a three‐electrode configuration with a CHI760E electrochemical workstation (Chinstruments, China). The SNOVA heterostructure film coated on the FTO served as the working electrode, while platinum (Pt) acted as the counter electrode and Ag/AgCl in KOH solution was used as the reference electrode. A 980 nm continuous‐wave laser (MDL‐XF‐980, CNI) served as the excitation source, modulated to a pulsed light at 0.2 Hz with power of 1.0 W, and a pulsed light at 0.5 Hz with power of 0.8 W. Meanwhile, steady‐state surface photoelectric voltage measurements were performed using PL‐SPV/IPCE1000 (PerfectLight, China) over a wavelength range of 300 to 1100 nm.

### In Vitro Cell‐Attached Patch‐Clamp Recording Under NIR Stimulation

Fresh brain tissue from mice (4 weeks) was sectioned at 300 µm thickness using a vibratome at 4 °C. The brain slices were immediately transferred to oxygenated artificial cerebrospinal fluid (aCSF) and incubated at 32 °C for 1 h. The composition of the oxygenated aCSF was as follows: 125 mmol NaCl, 2.5 mmol KCl, 1 mmol MgCl_2_, 2 mmol CaCl_2_, 11.1 mmol glucose, 1.25 mmol NaH_2_PO_4_, and 25 mmol NaHCO_3_. Following incubation, the brain slices were transferred to the recording chamber. Under bright‐field illumination with a 10× objective, the target brain region was identified. Subsequently, 5 µL of SNOVA solution (100 mg mL^−1^), UCNP suspension (100 mg mL^−1^ in PBS), or PBS was applied onto the slice. A 40× water immersion objective was then used to locate neurons in close proximity to SNOVA particles. Neurons were selected for recording based on healthy morphology, including a bright, rounded soma with clear boundaries. Electrophysiological recordings were performed in current‐clamp mode using an AxoClamp 700A amplifier (Axon, USA). A gentle positive pressure was applied to the pipette before immersion into the solution. After entry, the pipette was electrically compensated to maintain a potential near 0 mV. As the pipette approached the tissue, it was advanced at a slower rate while gently clearing the surrounding tissue to expose the target cell. The pipette tip was positioned on the upper‐right edge of the selected neuron, producing a slight resistance increase of ≈0.2–0.3 MΩ. Mild negative pressure was then applied to allow loose attachment of the pipette to the cell membrane. When the seal resistance reached 100–300 MΩ, cell‐attached patch‐clamp recordings were performed under current‐clamp mode (I = 0). At 10 s after the start of recording, NIR stimulation was applied using a 980 nm pulsed laser (0.5 Hz, 300 ms pulse width, 0.04 W/0.19 W/0.33 W/0.49 W/0.65 W).

### Vertebrate Animals Subjects

This study utilized adult male C57BL/6J mice (20–30 g, 8–10 weeks old, sourced from Phenotek Biotechnology Co.). All animal experimental procedures were approved by the ethical approval from the Ethics Committee for Animal Management at the Phenotek Biotechnology (approval number AUP‐20250515‐01). Mice were housed in groups under a 12‐h light/dark cycle (temperature: 20–25 °C, humidity: 50–65%) with food and water provided ad libitum.

### NIR Neurostimulation in the M2

This study involved mice that received injections of SNOVA solution, UCNP solution, or PBS into the M2 region (AP: +1 mm, ML: ±0.78 mm, DV: −1 mm). A total of 3 µL of solution was injected at 0.5 µL min^−1^ using a microsyringe pump (Pump 11 Elite, Harvard Apparatus) with a 33‐gauge needle. The needle should stay in the located brain tissue for 10 min to insure the full diffusion of the solution before withdrawal. All animals were allowed to fully recover for 24 h after the stereotaxic injection, during which they exhibited normal behavior before behavioral experiments commenced. It has been reported that a 12‐h postoperative recovery period was sufficient for studying neuromodulation‐induced behavioral changes with minimal interference from pain or anesthesia.

SNOVA heterostructures were dispersed in a sterile PBS to prepare a solution with a concentration of 100 mg mL^−1^ SNOVA/PBS. Prior to surgery, all surgical subjects were disinfected with 70% ethanol and rinsed with sterile deionized water and sterile PBS. Mice were then anesthetized with isoflurane, and hair was shaved from their heads using a blade. The exposed scalp was disinfected with povidone‐iodine. Erythromycin ointment was applied to both eyes to keep the ocular surface moist and protect against light exposure during the surgery.

The mice were placed in a stereotaxic frame (RWD), and their heads were secured. Surgical scissors were used to fully expose the skull without cutting any skin. A 3% hydrogen peroxide solution was employed to remove the underlying mucosa. For each animal, a burr hole with a diameter of 0.5 mm was drilled into the target brain region according to the specified anteroposterior (AP) and mediolateral (ML) coordinates. A total of 3 µL of the SNOVA/PBS mixed solution, UCNP/PBS solution (100 mg mL^−1^), or PBS solution was injected into the M2 region of the brain at a rate of 0.5 µL min^−1^ using a microinjection pump (Pump 11 Elite, Harvard Apparatus). Following the injection, the syringe was left in the brain tissue for 10 min to ensure adequate diffusion of the nanoparticles.

The fiber optic holder used to secure the mouse's head was green, allowing a custom Python program to track the mouse's movement trajectory. The fiber optic was inserted into the light fixture to ensure it was perpendicular to the mouse's head. In each experiment, a mouse was placed in a 30 × 30 cm black experimental box, allowing it to explore freely. A camera (Rebel T6, Canon) records video of the mouse for 10 s before, during, and 10 s after NIR photostimulation (640 × 480 pixels, 25 fps). During NIR‐I photostimulation, a 980 nm laser (MDL‐XF‐980, CNI) was emitted in pulsed mode from the fiber tip, with a frequency of 4 Hz and a pulse width of 150 ms. The laser illuminates the mouse's M2 region at power levels of 0.8 W for a duration of 10 s.

### NIR Neurostimulation in the VTA

All surgical procedures were conducted using a stereotaxic frame (RWD Life Science Co., Ltd.), with eight‐week‐old wild‐type C57BL/6J mice anesthetized via isoflurane inhalation delivered through a desktop anesthesia system (RWD). A scalp incision and craniotomy were performed over the VTA, followed by drilling a 5 mm‐diameter circular opening in the skull. A total of 3 µL of SNOVA suspension, pre‐sonicated for 5 min, was stereotactically injected into the VTA at 0.5 µL min^−1^ using a microsyringe pump (Pump 11 Elite, Harvard Apparatus) with a 33‐gauge needle. After injection, the needle was retained for 10 min before slow withdrawal to prevent backflow. A circular glass coverslip (5 mm diameter) was positioned over the craniotomy and secured with 3 M VetBond tissue adhesive, forming a stable, transparent window. A 3D‐printed fiber‐optic holder was aligned with the coverslip and fixed in place with additional dental cement. A NIR‐transmitting optical fiber (200 µm diameter) was inserted into the holder and positioned 2 mm above the skull surface, targeting the VTA injection site.

Two days after surgery, animals were habituated to a custom‐designed Y‐maze for a 30‐min pre‐experiment, followed by a 30‐min training session. During the three‐day training period, one arm of the maze was designated for light stimulation. Upon mouse entry into this arm, a programmed NIR pulse train was triggered. The setup involved two cameras: Camera 1 for real‐time object detection and Camera 2 for video acquisition. Image frames from Camera 1 were processed using Python. One maze arm was defined as the region of interest (ROI), which was converted to grayscale, binarized (threshold = 30), and inverted. Contours were extracted using cv2.findContours, and those with area >60 were identified as the mouse.

Once detected, a data acquisition (DAQ) card (USB‐6009, National Instruments) triggered a 980 nm NIR laser (MDL‐980) to deliver 150 ms pulses at 4 Hz (peak power: 0.8 W) transcranially through the skull.

Following three days of training, mice underwent a 30‐min testing phase under the same stimulation conditions. Recorded videos (Camera 2) were processed post hoc. The ROI was manually selected frame‐by‐frame, and black mouse contours were extracted using identical parameters to those applied during real‐time detection. Each frame was transformed to HSV color space, and a calibrated threshold was applied to isolate the mouse mask. Contours within area constraints were retained, and centroids were calculated to derive positional trajectories.

The tracked position data were then analyzed. X and Y coordinates were extracted, and the maze was divided into spatial grids. Time spent in each grid was normalized to the total recording time to create a frequency distribution. A Gaussian filter was applied to smooth the resulting matrix, and the final heatmap was rendered using the matplotlib library, with color intensity representing relative activity frequency.

### Real‐Time In Vivo Measurement of Dopamine Release during NIR Neuromodulation

A volume of 3 µL of SNOVA solution (prepared by dispersing 10 mg SNOVA in 100 µL PBS), UCNP solution (10 mg UCNPs in 100 µL PBS), or PBS alone was unilaterally injected into the VTA of C57BL/6J mice (8–10 weeks old). Seven days post‐injection, real‐time dopamine release was recorded using an in vivo i–t (current–time) electrochemical measurement. For the procedure, mice were anesthetized with 1.5 g kg^−1^ urethane dissolved in sterile saline via intraperitoneal injection and placed in a stereotaxic apparatus. A carbon fiber working electrode was implanted into the ventral striatum (coordinates: AP +1.3 mm, ML +0.8 mm, DV −4.2 mm). A silver wire auxiliary electrode and an Ag/AgCl reference electrode were inserted into the contralateral cortical hemisphere. A fiber optic cable for NIR light delivery was positioned 2 mm above the cranial window over the VTA. Electrochemical recordings were conducted using a CHI760E electrochemical workstation (Chenhua, Shanghai). A constant potential of +0.25 V versus Ag/AgCl was applied to the carbon fiber electrode, with data sampled at 0.1‐s intervals. To assess the capability of NIR transcranial stimulation in triggering dopamine release, NIR light pulses (140 ms pulse width, 1 Hz frequency, peak power 1.5 W) were delivered during the i–t recording. A total recording duration of 1500 s was used, with light stimulation applied for 10 s each at 600, 900, and 1200 s from the start of data acquisition.

### Immunohistochemical

For the c‐Fos expression analysis, mice were subjected to the turning task and Y‐maze preference test, followed by light stimulation targeting either the M2 or VTA region. Within 90 min post‐stimulation, mice were deeply anesthetized and transcardially perfused with 1× PBS, followed by 4% paraformaldehyde (PFA) in PBS. Brains were carefully dissected and post‐fixed in 4% PFA at 4 °C for 48 h to ensure thorough fixation. The tissues were then dehydrated, embedded in paraffin, and solidified by cooling at −20 °C on a freezing stage. Coronal brain sections (5 µm thick) were obtained from paraffin‐embedded tissues using a microtome (RM2016, Leica). Sections were deparaffinized and rehydrated through a graded ethanol series, followed by antigen retrieval in sodium citrate buffer (pH 6.0) heated to sub‐boiling temperature. To block nonspecific binding, sections were incubated with 3% bovine serum albumin (BSA) at room temperature for 30 min. Primary antibody incubation was performed overnight at 4 °C using an anti‐c‐Fos antibody (ab208942, Abcam). After primary incubation, slides were washed three times in 1× PBS and incubated with species‐specific fluorescent secondary antibodies for 50 min at room temperature in the dark. Nuclear staining was carried out with DAPI solution for 10 min under light‐protected conditions, followed by final PBS washes. Fluorescent images were acquired using a digital slide scanner (Pannoramic MIDI; 3DHISTECH). For quantification, brain sections from at least three animals per group were collected, imaged, and analyzed. All image acquisition and data analysis were performed in a double‐blinded manner.

### Evaluation of the Stability of SNOVA In Vivo

SNOVA solution (3 µL, 100 mg mL^−1^) was injected into the VTA of C57BL/6J mice as described above. The animals were killed 1 day, 1 week, 2 weeks, and 4 weeks after the injection, and 60‐µm‐thick acute coronal sections containing the injected SNOVA near the VTA were collected from the freshly dissected brains. The acute coronal sections were analyzed using a confocal microscopy (TSC SP8 STED, Leica) with an excitation wavelength of 405 nm, and the emission peak of CsPbBr_3_ at 483 nm.

### Evaluation of the Biocompatibility of SNOVA In Vivo

A total of 3 µL SNOVA solution (100 mg mL^−1^) was stereotactically injected into the VTA of C57BL/6J mice following the previously described protocol. At 1 day, 1 week, 2 weeks, and 4 weeks post‐injection, animals were anesthetized and perfused transcardially—initially with 1× PBS, followed by 4% paraformaldehyde (PFA) in PBS. The brains were carefully extracted and further immersed in 4% PFA at 4 °C for 48 h to ensure complete fixation. After fixation, the tissues underwent dehydration and paraffin embedding. Solidification was completed by placing the blocks on a freezing stage at −20 °C. Paraffin‐embedded brains were sectioned coronally into 5‐µm‐thick slices using a cryostat (RM2016; Leica). Tissue sections were deparaffinized and rehydrated through graded ethanol series, followed by antigen retrieval using sodium citrate buffer (pH 6.0) heated to a sub‐boiling temperature. To prevent nonspecific binding, the slices were blocked with 3% bovine serum albumin (BSA) at room temperature for 30 min. Primary antibody incubation was carried out overnight at 4 °C. The antibodies used for immunostaining included anti‐GFAP (E4L7M) XP Rabbit mAb #80 788, anti‐Iba‐1 (ab178846; Abcam), and anti‐caspase‐3 (#9662). After primary incubation, the slides were washed three times with 1× PBS and subsequently incubated with species‐specific fluorescent secondary antibodies for 50 min in the dark at room temperature. Nuclear staining was performed by adding DAPI solution for 10 min under light‐protected conditions, followed by final PBS washes. Fluorescence images were captured using a digital slide scanner (Pannoramic MIDI; 3DHISTECH). For quantification, brain slices from at least three animals per group were collected, imaged, and analyzed. All imaging and subsequent data analyses were conducted in a blinded manner with respect to treatment conditions.

### Assessment of Mouse General Health After SNOVA Injection

Ten male C57BL/6 mice (8 weeks old) were randomly assigned to the control group (n = 5, receiving 5 µL of PBS) or the SNOVA group (n = 5, receiving 5 µL of 100 mg mL^−1^ SNOVA). Stereotaxic injections were targeted to the left M2 cortex using standard coordinates (AP +1.0 mm, ML +0.78 mm, DV −1.0 mm) with an infusion rate of 0.5 µL min^−1^. To minimize reflux, the injection needle was retained in place for 10 min following infusion. Body weight was recorded on the day before surgery (baseline), the day of surgery, and on postoperative days 3, 7, 10, 13, 16, 19, 26, and 33. Locomotor activity was assessed at the same time points using a 10‐min Open Field Test (OFT). Video recordings from the OFT were analyzed with a custom Python script that segmented the darker mouse from the lighter background, localized the animal's centroid on a frame‐by‐frame basis, and reconstructed locomotor trajectories. All body‐weight and locomotor data were analyzed in GraphPad Prism 10. Results were presented as mean ± SD with individual data points (scatter). Between‐group differences at each time point were evaluated by multiple t‐tests.

### Statistical Information

The firing frequency of neurons (Figure 3), linear displacement in behavioral assays (Figure 4), cumulative dopamine (DA) release (Figure 5), place preference (Figure 5), as well as cell density and population proportions (Figures 4 and 5) were quantified and statistically analyzed. Data analysis was performed using OriginPro 2021, Python (v3.11), PyCharm (v2024.1.4), and ImageJ (Win64, v1.54; NIH). All statistical evaluations were conducted in Origin 2021. The Shapiro–Wilk test was used to assess the normality of data distributions. For comparisons of neuronal firing frequency between two groups, unpaired t‐tests were conducted, and statistical significance was indicated as ^*^ *p* < 0.05, ^**^ *p* < 0.01, ^***^ *p* < 0.001 and ^****^ *p* < 0.0001. As the data for c‐Fos expression was normally distributed, these were analyzed using one‐way analysis of variance (ANOVA) followed by Tukey's post‐hoc multiple comparisons test. The data for linear displacement and cumulative DA release were analyzed using ANOVA without normality assumption, given its reasonable tolerance of violations to normal distribution.^[^ ^77^, ^78^ ^]^ For the place preference test, paired t‐tests were used to evaluate within‐group changes before and after training when the data conformed to normality. For the preference change data comparing all three groups, the data followed a normal distribution and, thus, one‐way ANOVA was used followed by Tukey's post‐hoc comparison test. Sample sizes were not determined by power analysis but were based on previous studies using similar behavioral and histological paradigms in the same brain regions. All analyses were conducted under blinded conditions.

### Estimation of Electric Field of SNOVA

Experimentally, 0.25 mg of SNOVA was uniformly spread over a 100 mm^2^ FTO substrate (S_FTO_) exposed to a 1 W 980 nm light spot of 1 mm^2^ (S_NIR_), the average photocurrent was measured as *I_FTO_* = 6.15 nA (Figure 3d). The photocurrent per milligram of SNOVA can be estimated as:

(1) $$\begin{array}{ccc} 6.15\mathrm{nA}/S_{\mathrm{NIR}}\times S_{\mathrm{FTO}}/0.25\mathrm{mg} & & =6.15\;\mathrm{nA}/1\;mm^{2}\times 100\;mm^{2}/0.25\mathrm{mg}\\ & & =2460\;\mathrm{nA}/\mathrm{mg} \end{array}$$

Given that 3 µL of SNOVA (100 mg mL^−1^, i.e., 0.3 mg) was injected in vivo, the resulting photocurrent generated by the injected SNOVA in vivo can be estimated as:

(2) $$I_{\mathrm{in}\;\mathrm{vivo}}=0.3\;\mathrm{mg}\times 2460\;\mathrm{nA}/\mathrm{mg}=738\;\mathrm{nA}$$

Now the electric field generated was estimated by the SNOVA in vivo, assuming the SNOVA aggregate within a spherical region of radius *R_SNOVA_* ≈ 20 µm based on the Fluorescence image (Figure 6a). Outside the sphere (*r* > *R_SNOVA_*), the photocurrent can be approximated as a point current source, and the electric field decays with the square of the distance:

(3) $$E_{\mathrm{out}}\left(r\right)=\frac{I_{\mathrm{in}\;\mathrm{vivo}}}{4\pi \sigma r^{2}}$$

The maximum electric field is at *r* = *R_SNOVA_* as:

(4) $$E_{max}=E_{out}\left(r=20\mu m\right)=734\;\mathrm{mV}/\mathrm{mm}$$

Previous electrophysiological studies have shown that neuronal activation in acute brain slice preparations requires an electric field of approximately *E*_*thres*h*old*_ = 100 mV mm^−1^.^[^ ^79^ ^]^ Since *E_max_* exceeds the activation threshold *E*_*thres*h*old*_, neuronal activation was achievable.

Further, the radius was estimated within which range the electric field is sufficient to activate neurons as *E_out_* = *E*_*thres*h*old*_, the outer threshold radius is calculated as *r_threshold_* = 54.2 µm.

To take the power intensity into account, considering the 15 mm water layer between the glass tank sidewall of the Electrolytic cell and the FTO electrode, and an attenuation coefficient of water at 980 nm of ≈0.048 mm^−1^,^[^ ^80^ ^]^ the power at the FTO is reduced to about:

(5) $$P_{FTO}=1\times \exp\left(-1\times 15\times 0.048\right)=0.487\;W$$

Taking SNOVA located ≈2 mm beneath the brain surface example, and using an effective brain tissue attenuation coefficient of ≈0.5 mm^−1^,^[^ ^80^ ^]^ the effective light power illuminating on the SNOVA is:

(6) $$P_{VTA}=1\times \exp\left(-1\times 2\times 0.5\right)\times =0.368\;W$$

Therefore, the attenuated power remains at the same order and sufficient to support the estimated activation range.

## Conflict of Interest

The authors declare no conflict of interest.

## Author Contributions

L.J., C.M., and Y.Z. contributed equally to this work. L.S. and L.J. performed in conceptualization. L.S. and P.T. performed in project administration. L.S., P.T., and K.Z. performed in funding acquisition. L.S., P.T., L.J., and L.Q. performed in methodology. C.M. and L.J. performed in synthesis of nanoparticles. L.J., Y.G., Y.W., and Y.X. performed in optical measurements. L.J. and Y.Y. performed in photocurrent measurements. L.J. performed in confocal fluorescence microscopy and immunofluorescence. Y.Z., L.J., and C.M. performed in electron Imaging. L.J. and S.J. performed in patch‐clamp experiment. L.J., J.L., H.S., and Y.T. performed in in vitro and in vivo experiments. L.J., L.S., Y.Y., Y.Z., and P.T. performed in data analysis. L.J., G.L., Y.L., L.H., and P.C. performed in visualization. L.J. performed in writing – original draft. L.S., Y.Y., P.T., and L.J. performed in writing – review and editing. All authors discussed the results and commented on the manuscript at all stages.

## Supporting information



Supporting Information

## Data Availability

All data needed to evaluate the conclusions in the paper are present in the paper and/or the supplementary materials. Additional data related to this paper and the customized scripts are available from the corresponding author upon reasonable request.
