# Supplementary material for: NIR‐Triggered Upconversion‐Perovskite Heterostructures for Non‐Genetic, Implant‐Free Optoelectronic Neuromodulation
Source: Adv Sci (Weinh). 2025 Nov 23;13(8):e13844. doi: 10.1002/advs.202513844 (PMC12884713; doi:10.1002/advs.202513844)
Supplement: Supplementary file 1 — Supporting Information [file ADVS-13-e13844-s001.docx]

**Supplementary Materials for**

**Title - NIR-Triggered Upconversion-Perovskite Heterostructures for Non-genetic, Implant-Free Optoelectronic Neuromodulation**

*Luyue Jiang, Chenguang Ma, Yiping Zhao, Jiazhi Li, Gen Li, Shuang Jin, Haoyang Su, Ye Tian, Yingkang Yang, Yunfu Luo, Lei Huang, Peijie Chen, Yiming Gao, Yi Wei, Yike Xiang, Lunming Qin, Kaihuan Zhang, Yifei Ye*, Pengyi Tang*, Liuyang Sun**

L. Jiang, C. Ma, J. Li, G. Li, S. Jin, H. Su, Y. Tian, Y. Yang, Y. Luo, L. Huang, P. Chen, Y. Gao, Y. Wei, Y. Xiang, K. Zhang, Y. Ye, L. Sun

State Key Laboratory of Transducer Technology, Shanghai Institute of Microsystem and Information Technology, Chinese Academy of Sciences, Shanghai 200050, China

E-mail: [yeyifei@mail.sim.ac.cn](mailto:yeyifei@mail.sim.ac.cn); [Liuyang.Sun@mail.sim.ac.cn](mailto:Liuyang.Sun@mail.sim.ac.cn)

L. Jiang, C. Ma, Y. Zhao, J. Li, G. Li, S. Jin, H. Su, Y. Tian, Y. Yang, Y. Luo, L. Huang, P. Chen, Y. Gao, Y. Wei, Y. Xiang, K. Zhang, Y. Ye, P. Tang, L. Sun

2020 X-Lab, Shanghai Institute of Microsystem and Information Technology, Chinese Academy of Sciences, Shanghai 200050, China

E-mail: [py.tang@mail.sim.ac.cn](mailto:py.tang@mail.sim.ac.cn)

G. Li, H. Su, Y. Tian, Y. Yang, P. Chen, Y. Gao, Y. Wei, Y. Xiang, K. Zhang, P. Tang, L. Sun

School of Integrated Circuits, University of Chinese Academy of Sciences, Beijing 100049, China

G. Li, H. Su, Y. Tian, Y. Yang, P. Chen, Y. Gao, Y. Wei, Y. Xiang, K. Zhang, L. Sun

State Key Laboratory of Transducer Technology, Shanghai Institute of Microsystem and Information Technology, Chinese Academy of Sciences, Shanghai 200050, China

L. Huang, L. Qin

College of Electronics and Information Engineering, Shanghai University of Electric Power, Shanghai 201306, China

P. Tang

National Key Laboratory of Materials for Integrated Circuits, Shanghai Institute of Microsystem and Information Technology, Chinese Academy of Sciences, Shanghai 200050, China

L. Jiang, C. Ma, Y. Zhao contributed equally.

**Table of Contents**

Figure S1 to S18 and Table S1 to S2.


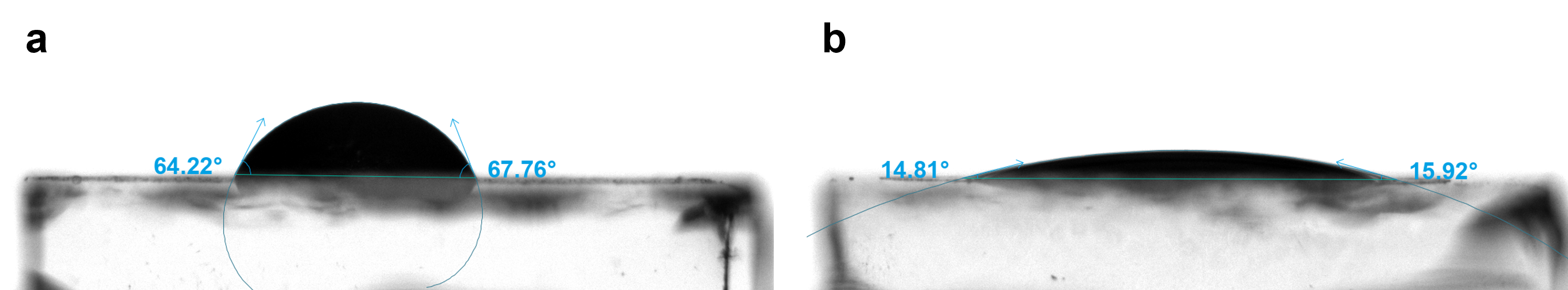


**Figure S1.** Static water contact angle tests of SNOVA without or without oleic acid (OA) removed. a) Static water contact angle of SNOVA without OA removed. b) Static water contact angle of SNOVA with OA removed.


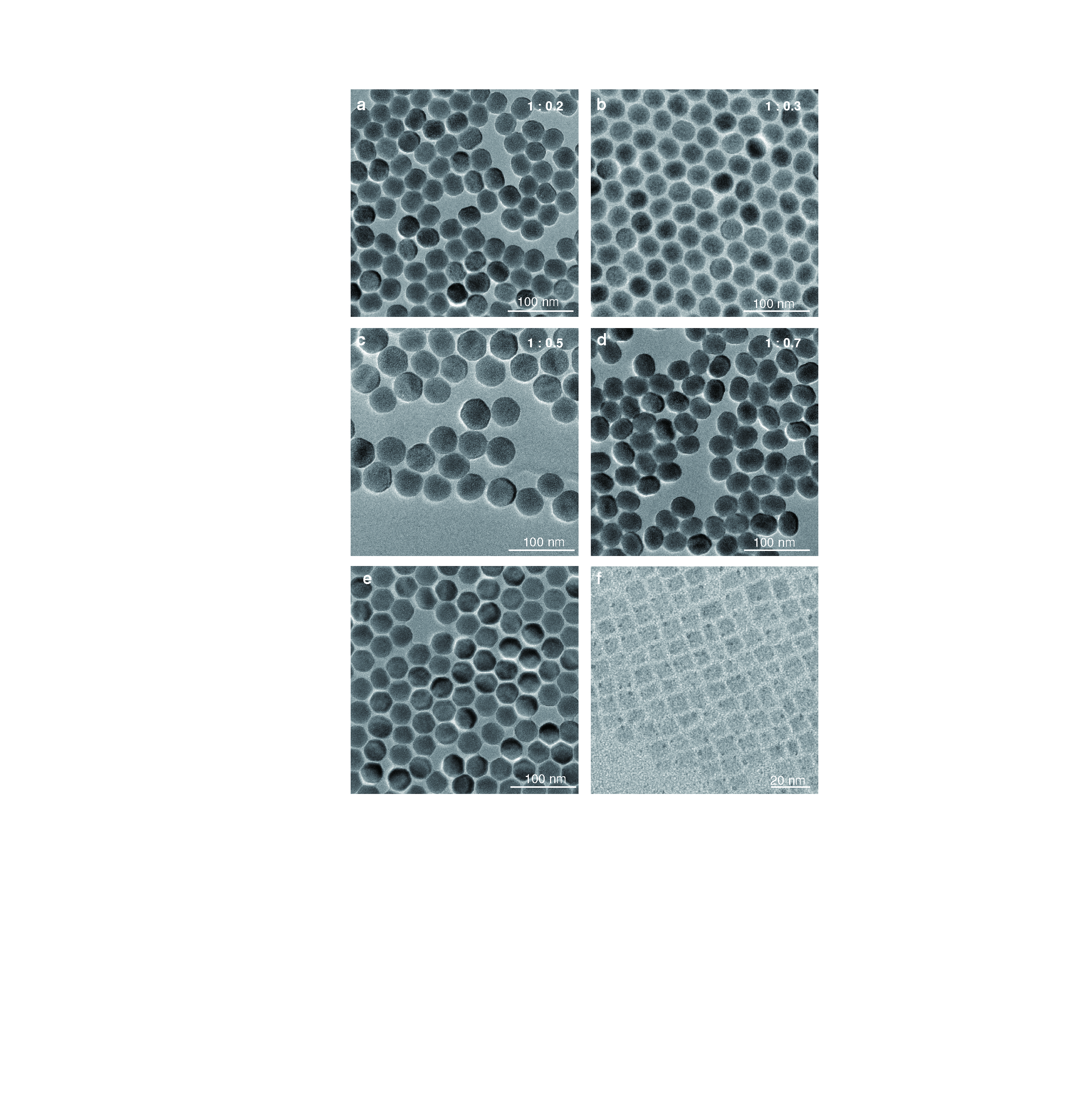


**Figure S2.** TEM images of different nanoparticles. a) TEM image of SNOVA with the input molar ratios of UCNPs to CsPbBr_3_ QDs of 1:0.2. b) TEM image of SNOVA with the input molar ratios of UCNPs to CsPbBr_3_ QDs of 1:0.3. c) TEM image of SNOVA with the input molar ratios of UCNPs to CsPbBr_3_ QDs of 1:0.5. d) TEM image of SNOVA with the input molar ratios of UCNPs to CsPbBr_3_ QDs of 1:0.7. e) TEM image of UCNPs. f) TEM image of CsPbBr_3_ QDs.

**
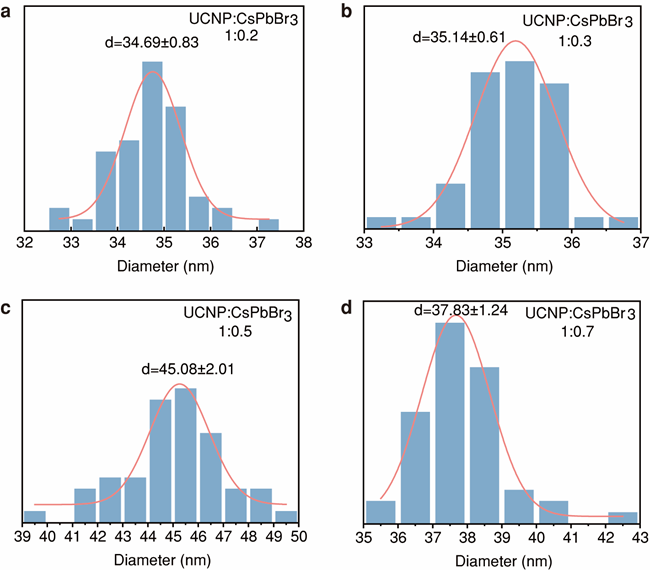
**

**Figure S3.** Particle size statistics of different nanoparticles. a) SNOVA with the input molar ratios of UCNPs to CsPbBr_3_ QDs of 1:0.2. b) SNOVA with the input molar ratios of UCNPs to CsPbBr_3_ QDs of 1:0.3. c) SNOVA with the input molar ratios of UCNPs to CsPbBr_3_ QDs of 1:0.5. d) SNOVA with the input molar ratios of UCNPs to CsPbBr_3_ QDs of 1:0.7.


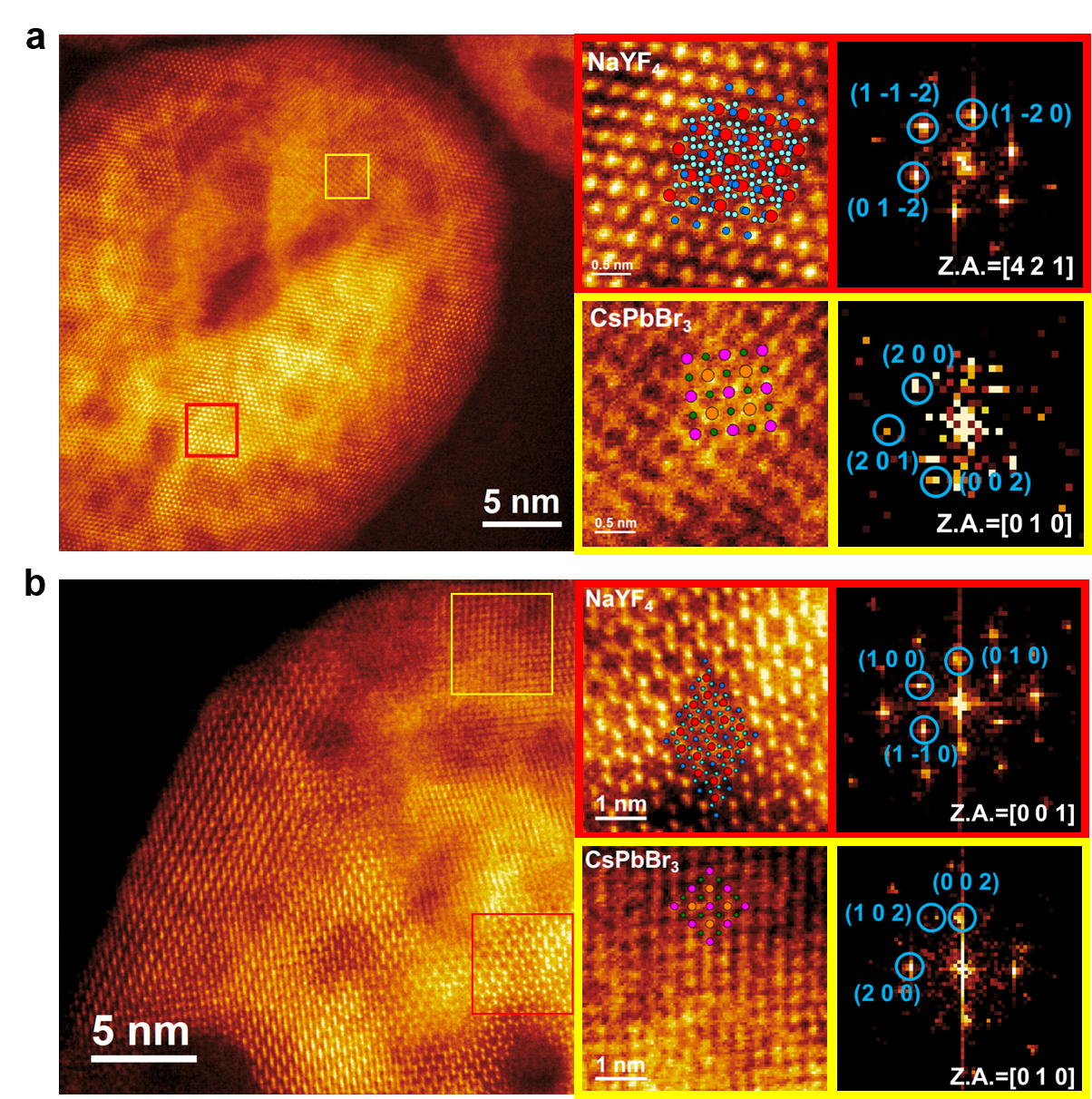


**Figure S4.** Aberration-corrected STEM-HAADF images of SNOVA after Wiener filtering. Enlarged views of the red- and yellow-boxed regions (with overlapping atomic models of hexagonal NaYF_4_:Yb/Tm UCNPs and cubic CsPbBr_3_ are shown, together with their corresponding FFT spectra. Y atoms are shown in red, Na in blue, F in cyan, Pb in pink, Cs in orange, and Br in green. NaYF_4_ viewed along the [421] zone axis and CsPbBr_3_ along the [010] direction.


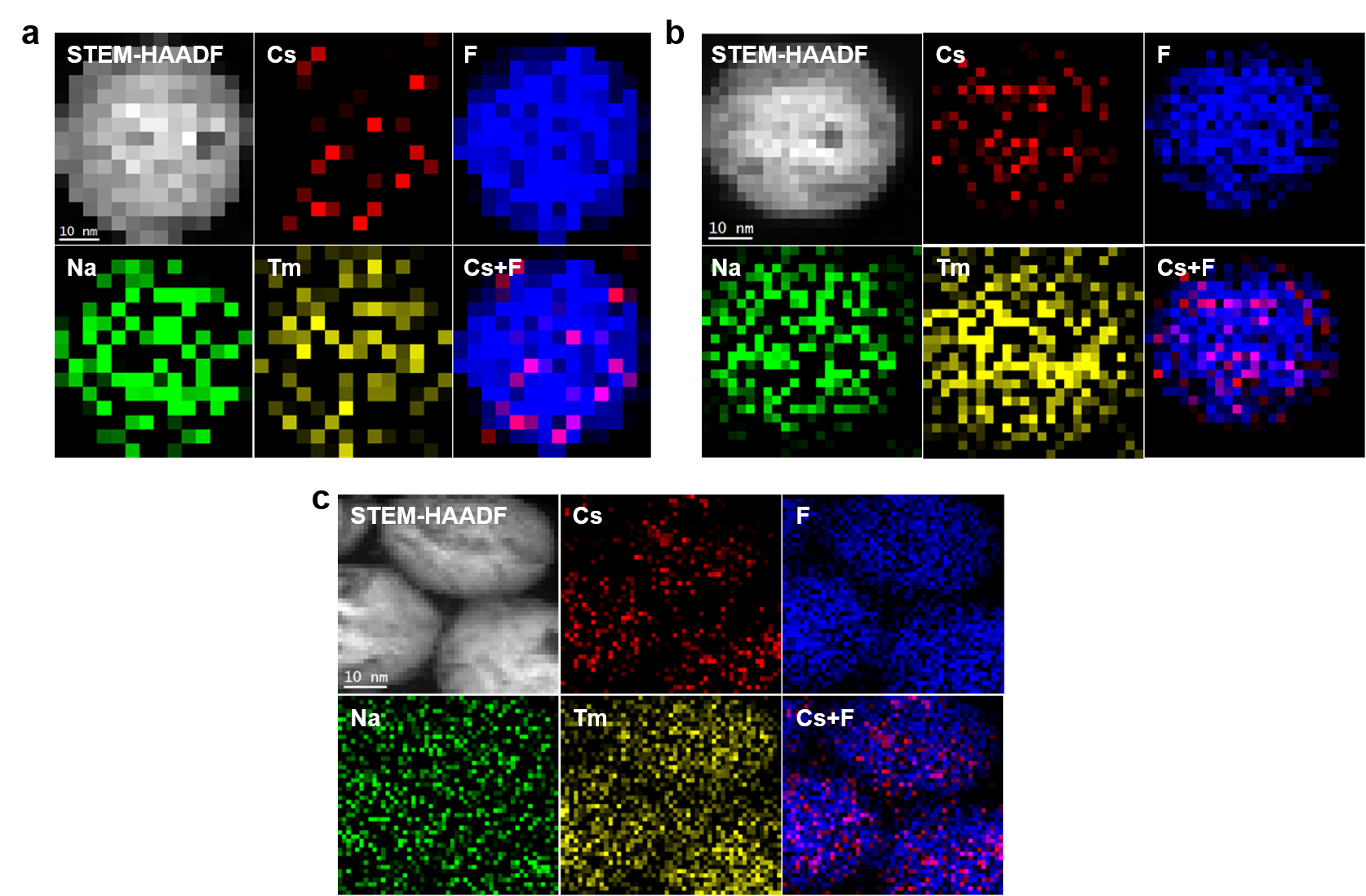


**Figure S5.** STEM-HAADF images and EELS elemental maps of SNOVA samples. Elemental distributions of Na, Tm, F and Cs demonstrate that CsPbBr_3_ is unevenly embedded within the NaYF_4_ matrix.

X-ray photoelectron spectroscopy analysis of SNOVA

From the Survey X-ray photoelectron spectroscopy (XPS) spectra, SNOVA had Na 1s, F 1s, Y 3d, Yb 4d, Tm 4d, Cs 3d, Pb 4f and Br 3d peaks (Supplementary Figure S6a). For SNOVA, the atom percentages of Na, F, Y, Yb, Tm, Cs, Pb, Br were 4.33 %, 34.99 %, 7.67 %, 0.64 %, 0.32 %, 0.68 %, 0.48 % and 2.44 %, respectively (Supplementary Table S2). Supplementary Figure S6b-d showed Pb 4f, Cs 3d and Br 3d spectra for SNOVA. The Pb 4f peaks for the untreated SNOVA sample contained separated features located at 143.14 and 138.28 eV, which are attributed to Pb^2+^ species from Pb^2+^−Br^−^ bonding in the perovskite. Similarly, in the XPS spectra of Cs 3d, a component associated with metallic Cs^0^ at 720.48 and 735.07 eV, and a component related to Cs^1+^ at 724.44 and 738.36 eV were identified. Br 3d spectra was shown in Supplementary Figure S6d. Well separated peaks from spin−orbit coupling for the original SNOVA powder showed two main peaks located at 67.96 and 68.95 eV.


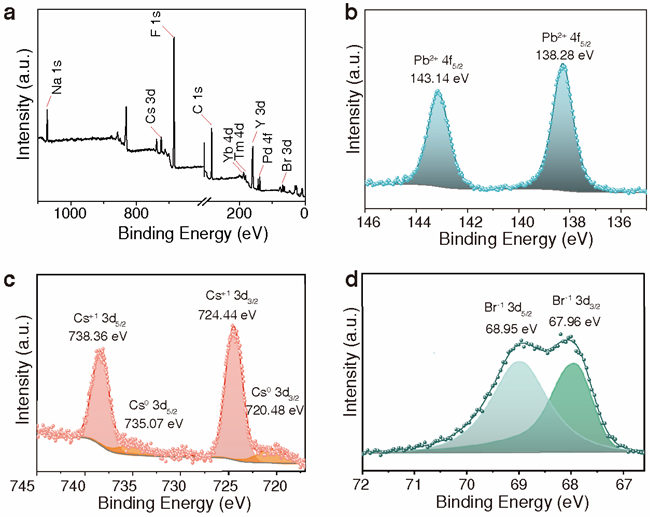


**Figure S6.** X-ray photoelectron spectroscopy (XPS) spectra of SNOVA. a) XPS survey spectra of SNOVA. High-resolution b) Pd 4f, c) Cs 3d, and d) Br 3d XPS spectra of SNOVA.


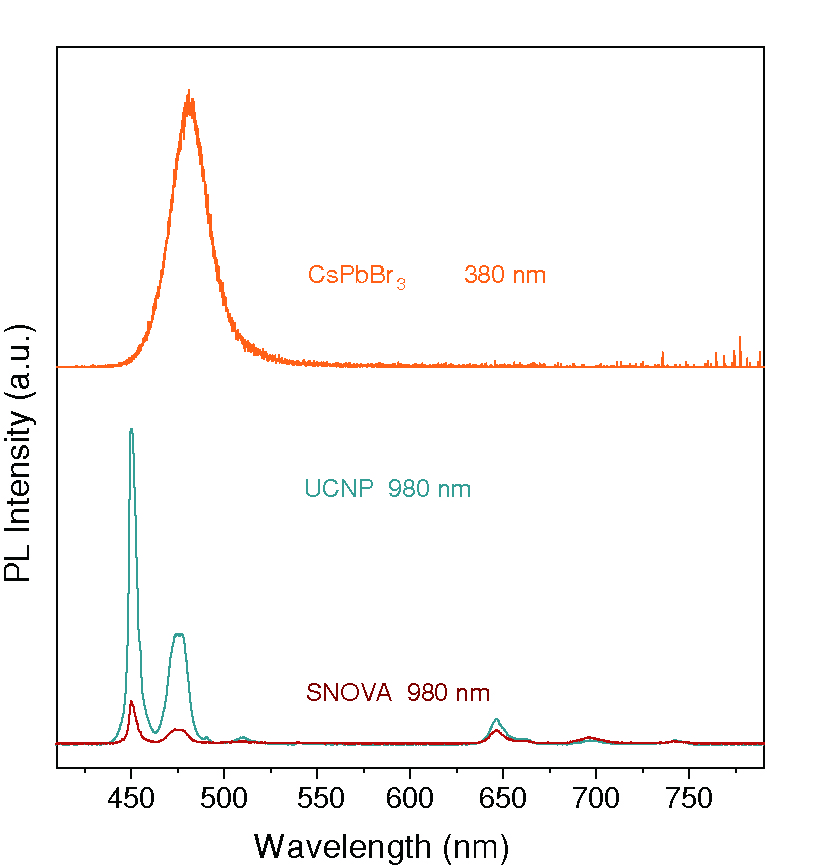


**Figure S7.** PL spectra of SNOVA, UCNPs and CsPbBr_3_ QDs. The PL spectra of SNOVA (red line) and UCNPs (blue line) were recorded under 980 nm excitation, while the PL spectrum of CsPbBr_3_ QDs (orange line) was collected under 380 nm excitation.


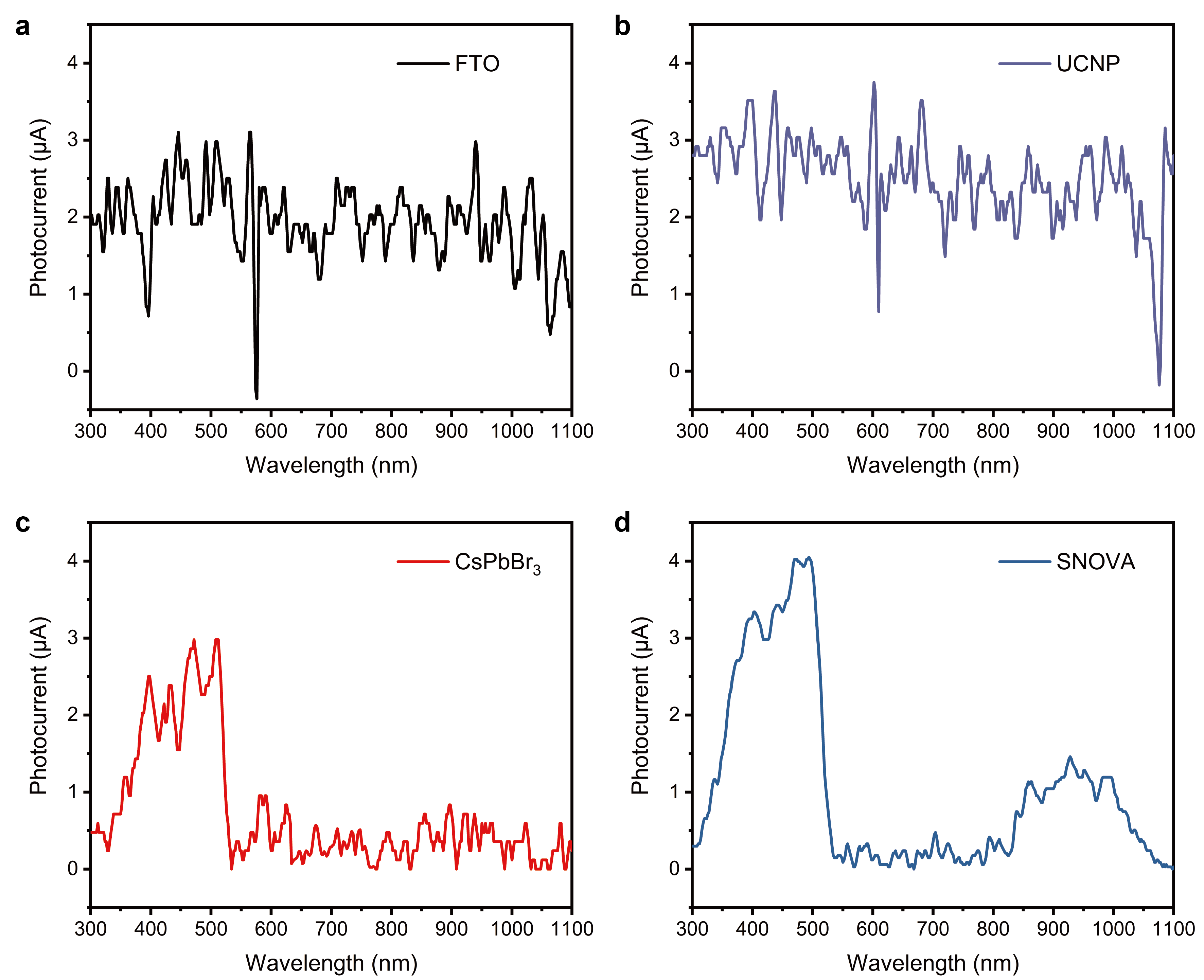


**Figure S8.** SPV spectra of a) FTO, b) NaYF_4_ UCNPs, c) CsPbBr_3_, and d) SNOVA.


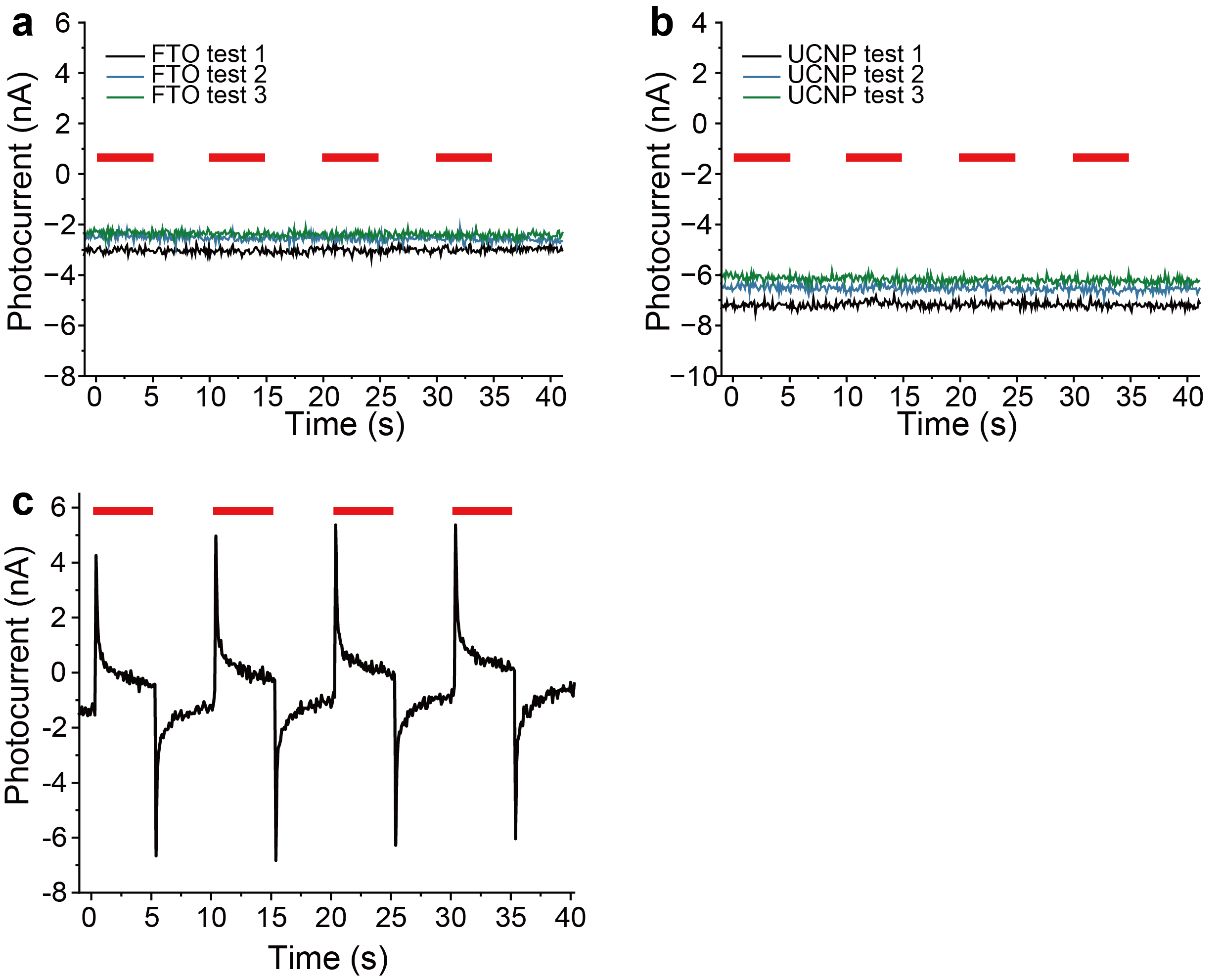


**Figure S9.** Time-dependent photocurrent (I-t) of a) pure FTO and b) FTO coated with pure NaYF_4_ UCNPs under 980 nm illumination (1 W) measured in PBS (pH 7.4). each sample was tested three times under identical conditions. Red bars indicate laser on (5 s per pulse).


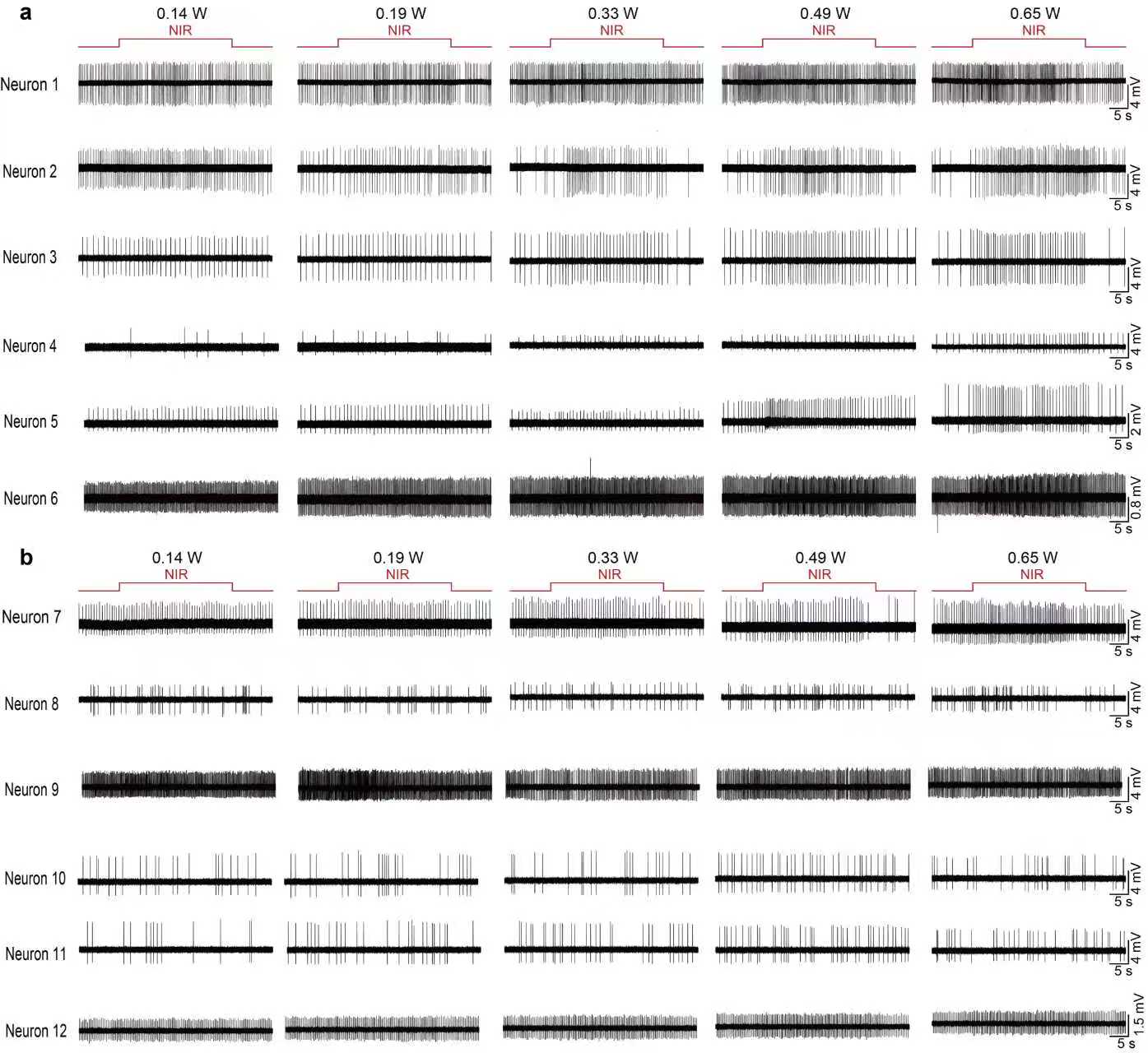


**Figure S10.** Cell-attached recordings of firing activity in VTA. a) Cell-attached recordings of firing activity in VTA dopamine neurons before and after NIR stimulation in an acute brain slice treated with SNOVA. b) Cell-attached recordings of firing activity in VTA dopamine neurons before and after NIR stimulation in an acute brain slice with PBS.


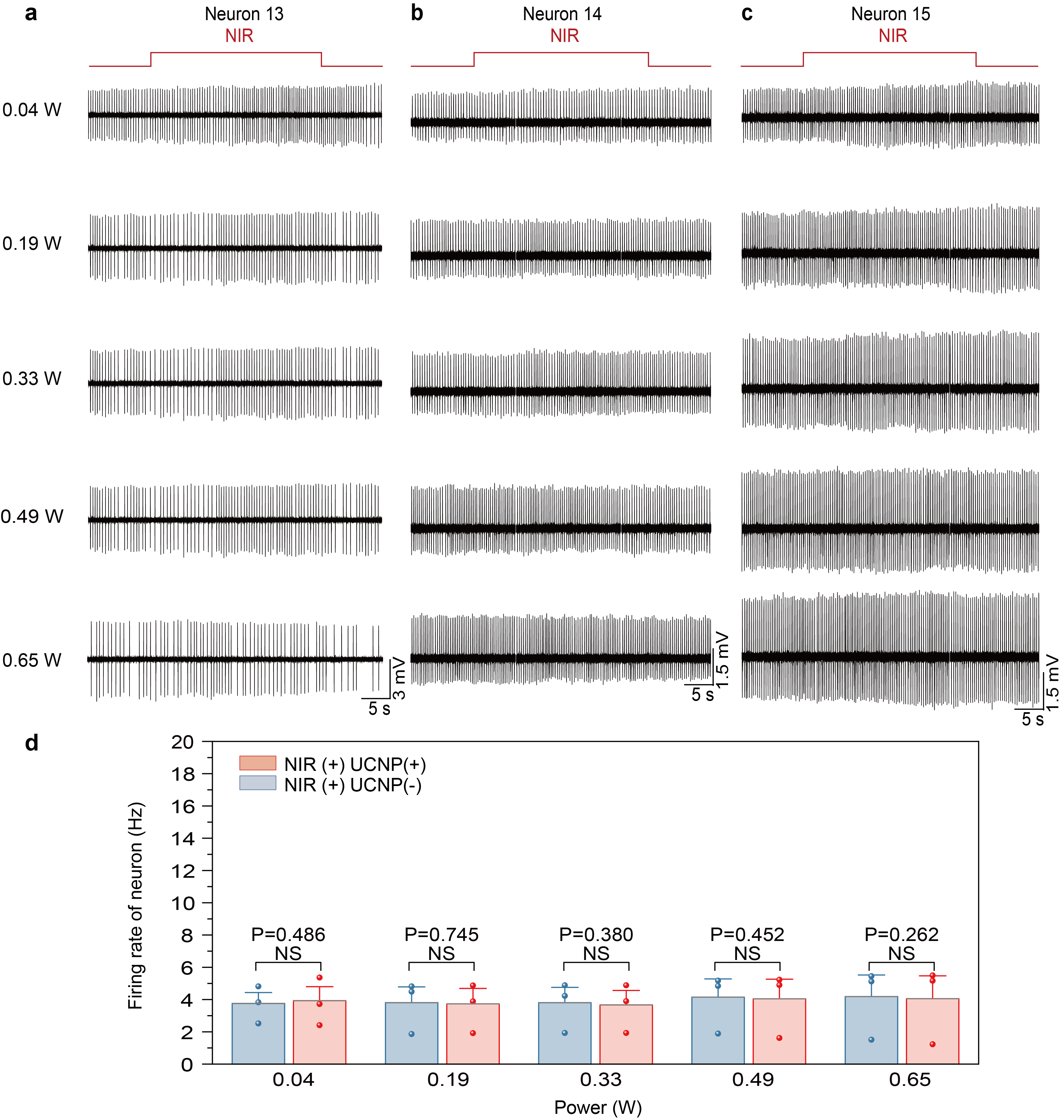


**Figure S11.** Cell-attached recordings of firing activity in VTA. a), b) and c) Cell-attached recordings of firing activity in VTA dopamine neurons before and after NIR stimulation in an acute brain slice treated with UCNP. d) Firing rate in VTA dopamine neurons under NIR stimulation in an acute brain slice treated with UCNP (n = 3 cells). Data are shown as mean ± SEM, with each point representing an independent trial; All groups followed a normal distribution, and pair-sample t-test was performed to calculate P values.


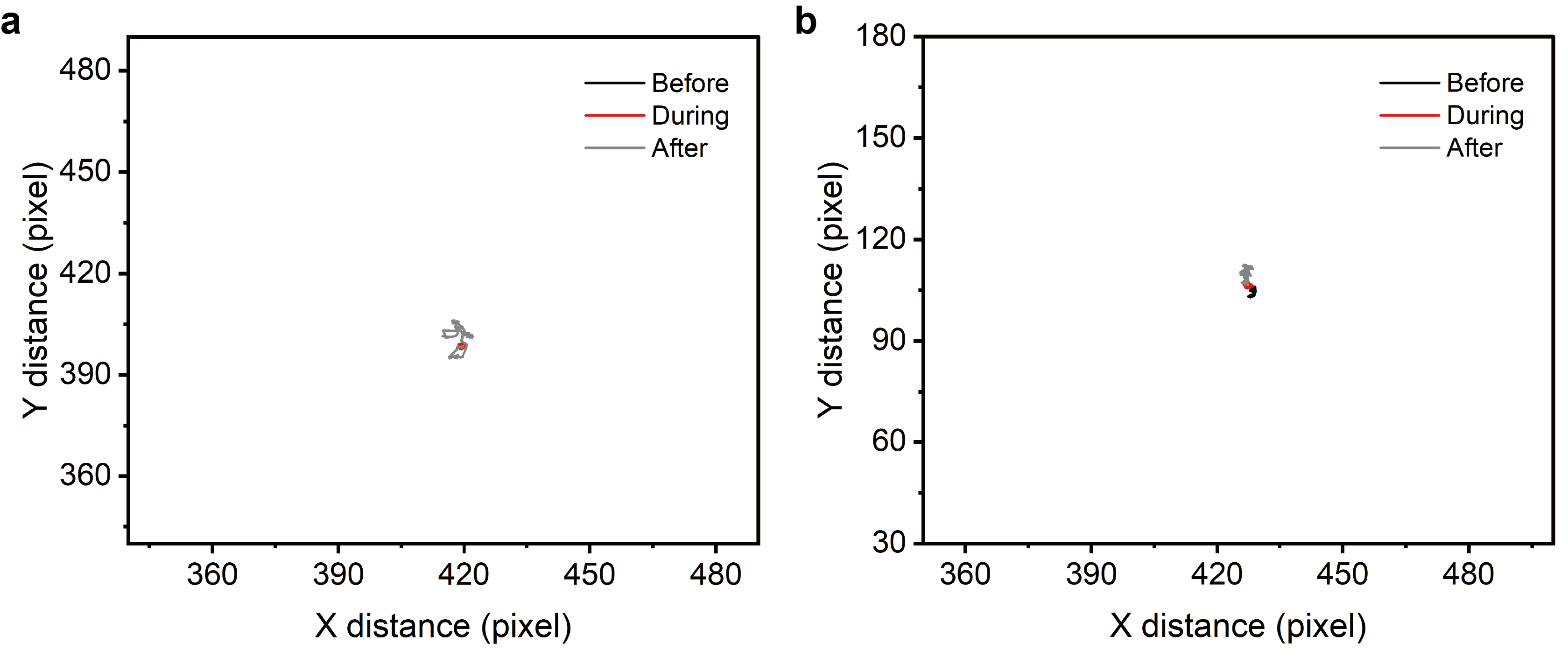


**Figure S12.** Locomotion trajectories of a) a PBS-treated mouse and b) a UCNP-treated control mouse before (black line), during (red line), and after (grey line) NIR stimulation of the M2 region.


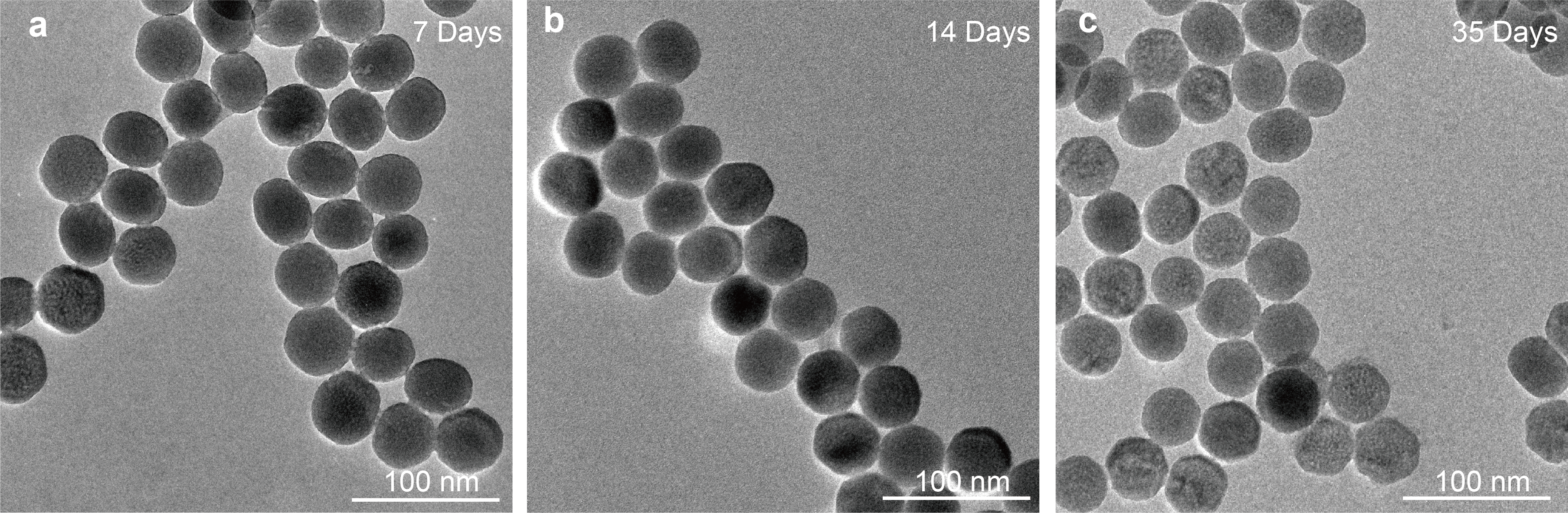


**Figure S13.** **TEM images of SNOVA heterostructures stored in DI water at room temperature.** a) 7 days, b) 14 days, and c) 35 days of immersion. The initial morphology prior to immersion is provided in Figure S2c.

­­
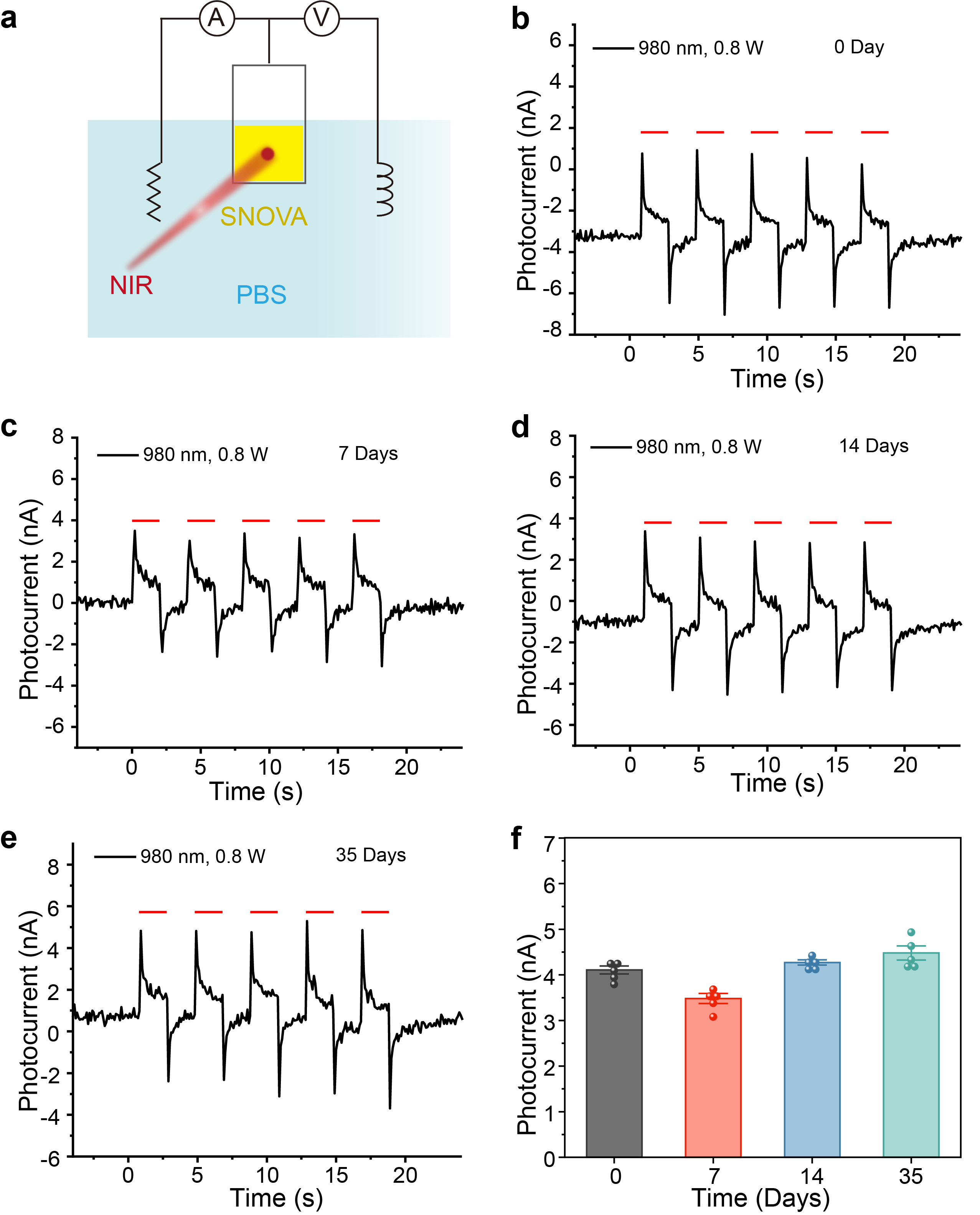


**Figure S14.** Photocurrent of SNOVA heterostructures stored in DI water over time. a) Schematic diagram of the three-electrode system for photocurrent measurements. b) photocurrent of SNOVA stored in DI water for 0 day. c) photocurrent of SNOVA stored in DI water for 7 days. d) photocurrent of SNOVA stored in DI water for 14 days. e) photocurrent of UCNP-CPBs stored in DI water for 35 days. f) photocurrent of SNOVA stored in DI water for different time. Data are shown as mean ± SEM.


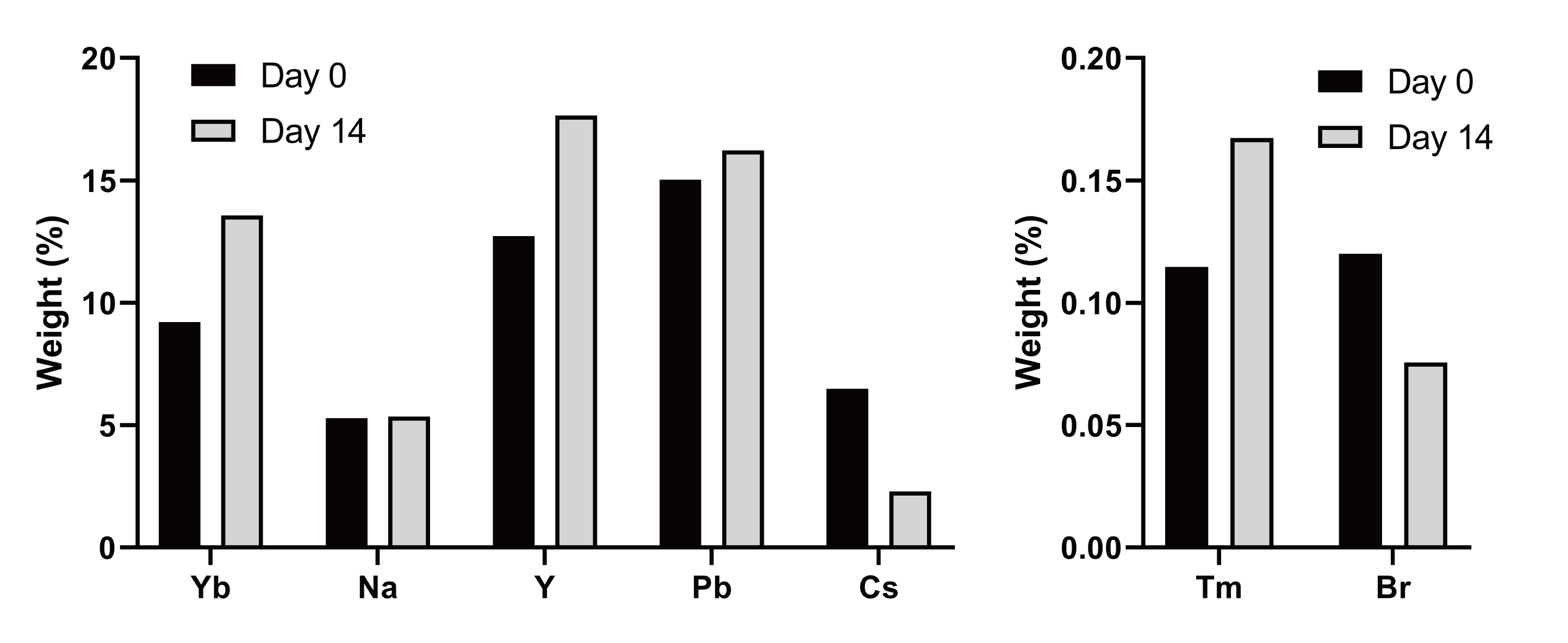


**Figure S15.** ICP-MS results showing weight ratios of Yb, Na, Y, Pb, Cs, Tm, and Br elements in SNOVA before and after immersion in PBS at 37 ℃ for 14 days.


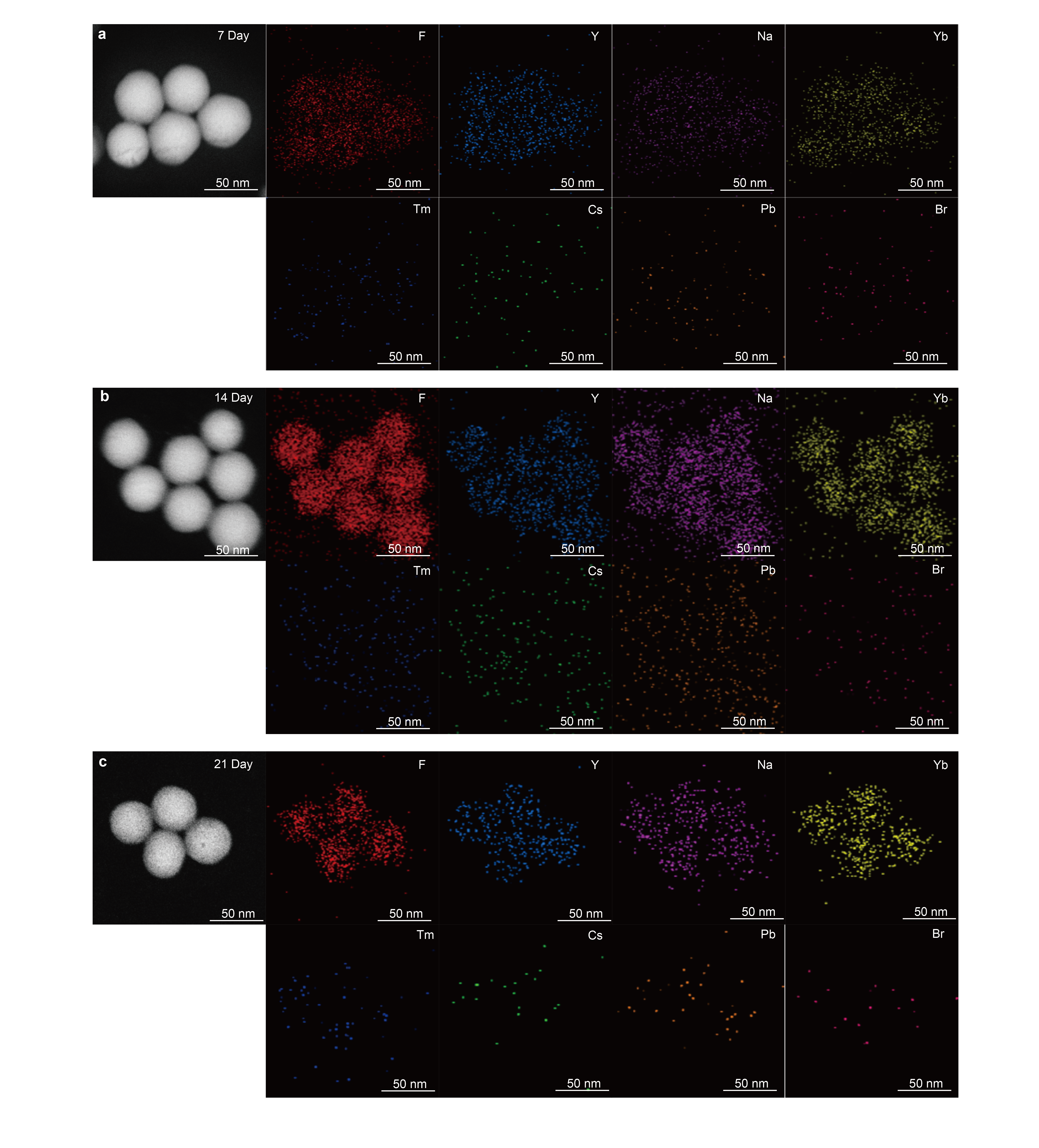


**Figure S16.** STEM-EDX images of SNOVA before and after immersion in PBS at 37 ℃ for 7, 14 and 21 days. Elemental maps of Yb, Na, Y, Pb, Cs, Tm, Br, and F confirm partial stability of the SNOVA heterostructure. Although a gradual reduction in particle size is observed (see Figure S17), the nanoparticles maintain overall morphology and elemental distribution, indicating mild surface dissolution without structural collapse or fragmentation.


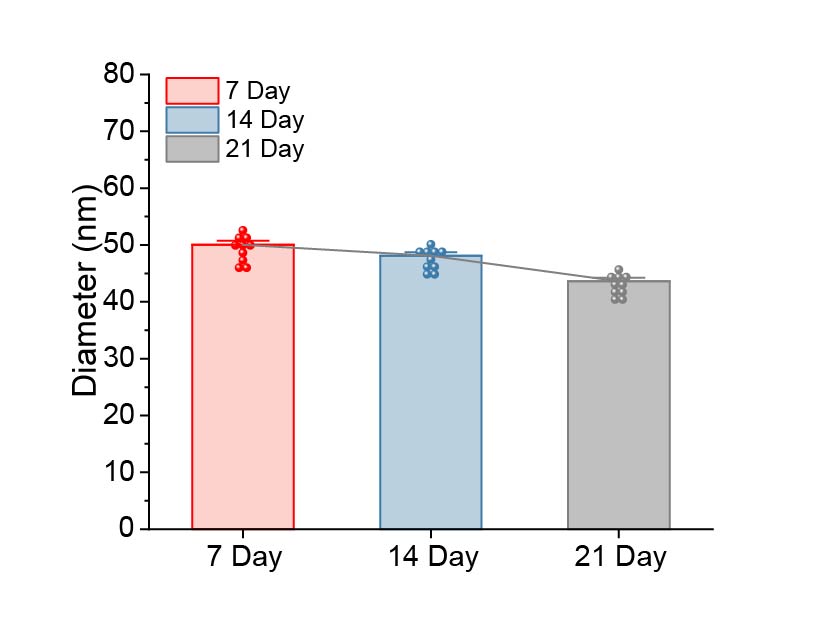


**Figure S17:** Time-dependent particle-size evolution of SNOVA in PBS at 37°C. The average diameters of SNOVA particles were analyzed from STEM images after immersion in PBS for 7, 14, and 21 days. A gradual decrease in particle size was observed, while the particles remained morphologically intact, indicating mild surface dissolution but preserved structural integrity.


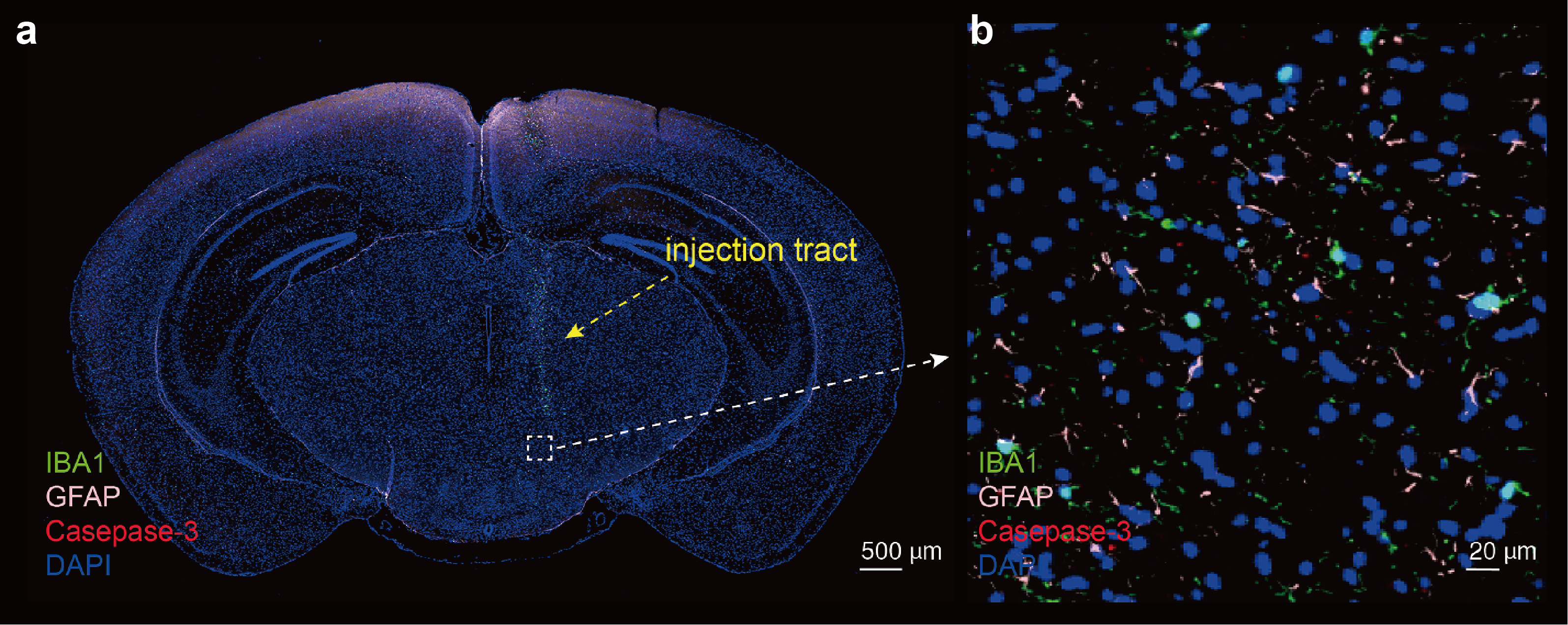


**Figure S18**. Confocal images showing immunofluorescence staining of the VTA at 4 weeks post-injection for PBS-injected control mice. Sections were labeled with Iba1 (green, microglia), GFAP (pink, astrocytes), Caspase-3 (red, apoptosis), and DAPI (blue, nuclei). a) Full view of the brain section. b) The enlarged view of the VTA region.


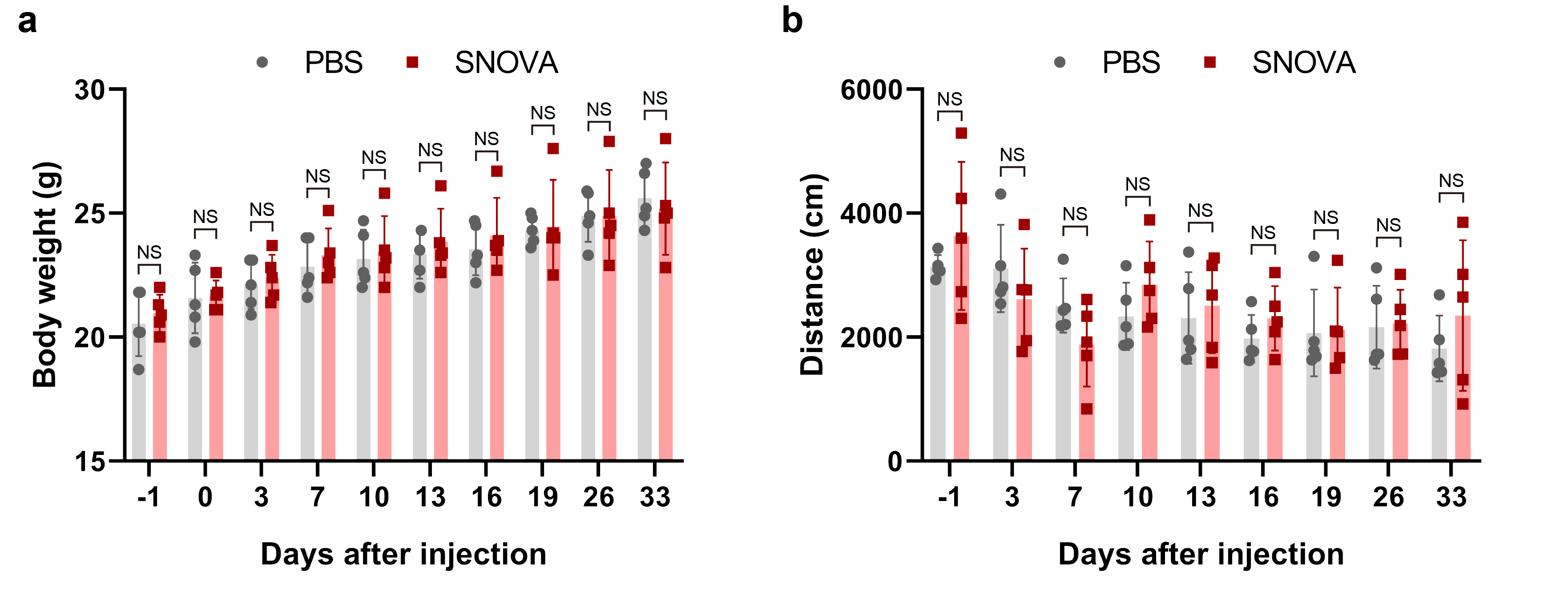


**Figure S19.** Assessment of mouse general health after SNOVA injection. a) Body weight of SNOVA-injected and PBS-injected control mice measured one day before surgery (-1), on the surgery day (0), and on postoperative days 3, 7, 10, 13, 16, 19, 26, and 33. b) Locomotor distance of SNOVA-injected and PBS-injected control mice measured one day before surgery (-1), and on postoperative days 3, 7, 10, 13, 16, 19, 26, and 33.

**Table S1.** The elemental composition of pristine SNOVA according to STEM-EDX

| Sample | F % | Y % | Na % | Tm % | Yb % | Cs % | Pb % | Br % |
| --- | --- | --- | --- | --- | --- | --- | --- | --- |
| SNOVA | 65.07 | 9.95 | 9.52 | 1.79 | 5.70 | 2.47 | 1.15 | 4.35 |

**Table S2.** XPS data analyses of the full survey scan spectra of the pristine SNOVA

| Sample | Cs 3d % | Pb 4f % | Br 3d % |
| --- | --- | --- | --- |
| SNOVA | 0.68 | 0.48 | 2.44 |
